# The Interaction of Vinculin with Actin

**DOI:** 10.1371/journal.pcbi.1002995

**Published:** 2013-04-25

**Authors:** Javad Golji, Mohammad R. K. Mofrad

**Affiliations:** 1 Molecular Cell Biomechanics Laboratory, Departments of Bioengineering and Mechanical Engineering, University of California, Berkeley, Berkeley, California, United States of America; 2 Physical Biosciences Division, Lawrence Berkeley National Lab, Berkeley, California, United States of America; University of California San Diego, United States of America

## Abstract

Vinculin can interact with F-actin both in recruitment of actin filaments to the growing focal adhesions and also in capping of actin filaments to regulate actin dynamics. Using molecular dynamics, both interactions are simulated using different vinculin conformations. Vinculin is simulated either with only its vinculin tail domain (Vt), with all residues in its closed conformation, with all residues in an open I conformation, and with all residues in an open II conformation. The open I conformation results from movement of domain 1 away from Vt; the open II conformation results from complete dissociation of Vt from the vinculin head domains. Simulation of vinculin binding along the actin filament showed that Vt alone can bind along the actin filaments, that vinculin in its closed conformation cannot bind along the actin filaments, and that vinculin in its open I conformation can bind along the actin filaments. The simulations confirm that movement of domain 1 away from Vt in formation of vinculin 1 is sufficient for allowing Vt to bind along the actin filament. Simulation of Vt capping actin filaments probe six possible bound structures and suggest that vinculin would cap actin filaments by interacting with both S1 and S3 of the barbed-end, using the surface of Vt normally occluded by D4 and nearby vinculin head domain residues. Simulation of D4 separation from Vt after D1 separation formed the open II conformation. Binding of open II vinculin to the barbed-end suggests this conformation allows for vinculin capping. Three binding sites on F-actin are suggested as regions that could link to vinculin. Vinculin is suggested to function as a variable switch at the focal adhesions. The conformation of vinculin and the precise F-actin binding conformation is dependent on the level of mechanical load on the focal adhesion.

## Author Summary

The interface between a cell and its substrate is strengthened by the formation of focal adhesions. In this study molecular dynamics simulations are used to explore the connectivity of one focal adhesion forming protein, vinculin, and the cytoskeletal filament, F-actin. The simulations demonstrate: (1) that vinculin can link along F-actin at these focal adhesions when it adopts an open conformation, (2) that the vinculin tail (Vt) can bind F-actin at its barbed-end preventing actin polymerization, (3) that vinculin can adopt two open conformations, and (4) that the second open conformation is necessary for vinculin to cap the actin filament. The results suggest that vinculin can act as a variable switch, changing its shape and the nature of its interaction with F-actin depending on the level of stress seen at a focal adhesion. Under the highest stress vinculin would adopt the open II conformation and link anywhere on F-actin, even its barbed-end. Under less stress vinculin could adopt the open I conformation and bind along F-actin. And under minimal stress vinculin could adopt its closed conformation. This variability allows for vinculin to truly function as the cell's mechanical reinforcing agent.

## Introduction

The focal adhesion is a critical for cell-substrate adhesions [1], [2], necessary for Cell movement [3], [4], wound healing [5], cancer cell metastasis [6], , and other processes [8]–[10]. At the focal adhesion, actin filaments of the cellular cytoskeleton are linked to the extra-cellular membrane (ECM) of the substrate [5]. Once linked, both mechanical forces [11] originating from within the cell – such as myosin induced contraction of the actin filaments [12] during cell migration – can act on the ECM and the substrate, and, mechanical forces originating from outside the cell – such as flow induced cyclic stress in the case of endothelial cells [13] – can be transduced to the cellular machinery [14].

Formation of the focal adhesion involves linkage of focal adhesion proteins to ECM bound integrins [15]–[18], linkage of focal adhesion proteins to each other [19], [20], and linkage of the ECM-focal adhesion complex to actin filaments [21]–[23]. The simplest focal adhesion complex would consist of a talin molecule bound to integrin via its head domain and bound to actin via its tail domain [24], [25]. Talin has 11 cryptic binding sites for vinculin and activation of these binding sites along with subsequent recruitment of vinculin to the growing focal adhesion correlates with the strengthening of the focal adhesion [26]. Recruitment of vinculin would reinforce the focal adhesion as vinculin can crosslink an actin filament to the talin molecule [27]. This binding of the focal adhesion to actin filaments, by vinculin or other focal adhesion forming molecules, is a critical step in completing formation of a mechanical link between the cell and its substrate.

**Figure 1:**
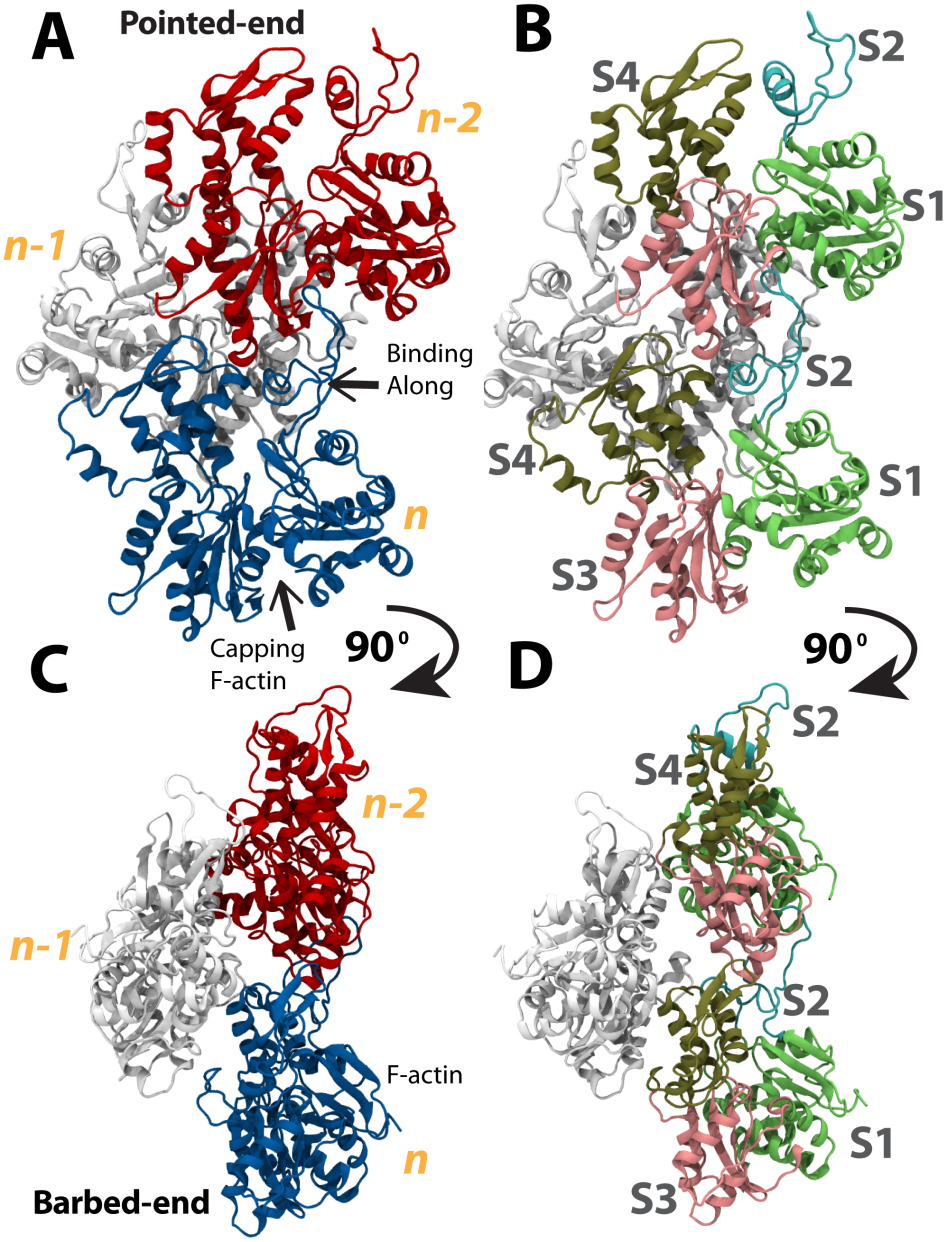
Structure of an actin filament. The actin filament is a polar double stranded polymer. Each subunit has a pointed (−) end and a barbed (+) end [28]. Actin filaments are formed mainly by addition of new actin subunits to the barbed (+) end. (A) The actin filament used in simulation for interacting with vinculin is three subunits in length: subunit *n* (blue), subunit *n-1* (white), and subunit *n-2* (red). The pointed-ends of all the subunits are aligned. An additional subunit *n-1* would add to the barbed-end of the filament. (B) Each actin subunit has four subdomains: S1 (green), S2 (cyan), S3 (pink), and S4 (tan). (C) View of the actin filament shown in (A) rotated 90 degrees. Actin subunit *n-1* lies behind *n* and *n-2* as viewed from (A). (D) View of the actin filament shown in (B) rotated 90 degrees. Each subunit of the actin filament is rotated relative to its neighboring subunits. This view shows the *n* subunit is rotated relative to the *n-2* subunit. Vinculin can bind actin at both along the filament and at the barbed end (arrows).

The actin filament itself is composed of numerous individual actin subunits bound together to form a polar double-stranded filament [28]. Multiple filaments can be crosslinked by actin crosslinkers [29], [30]. Both the actin subunits and the actin filament are polar, with a barbed-end (+) and a pointed-end (−). In this paper, the actin subunit at the barbed-end is referred to as subunit *n*, with the next subunit towards the pointed-end referred to as subunit *n-1*, and the subsequent subunit to *n-1* is referred to as *n-2*, and so on (Figure 1). Polymerization of the actin filament can occur at both ends of actin, but occurs with much higher efficiency at the barbed-end [31]. Each actin subunit has 4 subdomains: S1, S2, S3, and S4 [28]. The S2 subdomain contains a DNase-I-binding loop (D-loop) that can interact with a neighboring actin subunit [28]. Recently, it was shown that polymerization of F-actin at the pointed-end is slower than polymerization at the barbed-end because of an interaction between the D-loop at *n-1* with a hydrophobic patch of *n-2* (at the pointed-end) [32]. *n-1n-1*

Vinculin is a globular protein much smaller than actin and with 5 helical domains: domain 1 (D1), domain 2 (D2), domain 3 (D3), domain 4 (D4), and the vinculin tail domain (Vt) [33]. D1–D4 together form the vinculin head domains. With Vt bound to each of the vinculin head domains, vinculin is considered to be in a closed conformation. Vt contains the likely binding sites for an actin filament [34], while D1 contains the likely binding sites for talin [35]. In its closed conformation vinculin is unable to bind both F-actin at Vt and talin at D1 [36], and the closed conformation is often referred to as the auto-inhibited conformation [37]. D1 of the vinculin head inhibits the linkage of Vt with F-actin. Several hypotheses have been explored concerning the mechanism of vinculin activation [38]–[40]. It is clear that a vinculin conformational change is necessary to allow for binding of vinculin to both F-actin and talin [41]. A recent computational study has proposed a conformational change that could activate vinculin: D1 of the vinculin head could move away from Vt and towards its talin binding partner [42]. The movement of D1 leading to vinculin activation could result from a force-induced stretching of vinculin [40], [43] that would result from vinculin being recruited to mechanically stressed focal adhesions [43]. The computational studies capture one explanation for reinforcement of the linkage between a cell and its surface. Other studies examine additional possibilities such as force-responsive linkage to the substrate via syndecan-4 [44].

Vinculin recruitment to focal adhesions is made possible by activation of the vinculin binding sites (VBS) within the talin rod [45], [46]. The VBS are hydrophobic in nature and buried within the helical structure of talin in the absence of force [47]. Initial computational investigation [48]–[50] and later experimental investigation [45] has demonstrated the exposure of the buried hydrophobic VBS only after force induced stretch of the talin rod. Once activated, the VBS can bind D1 of vinculin. Recent computational simulation suggests the interaction of D1 with VBS is completed, again, after force-induced activation of vinculin [51]. The focal complex serves to link ECM-bound integrin to actin filaments: force-induced activation of talin and activation of vinculin are effective only if the activated vinculin can bind along the actin filament and link it to the focal adhesion. Binding of vinculin to F-actin is the final step to complete this structure.

Vinculin-actin binding is necessary for focal adhesions to be mechanically resilient [52], and the interaction is crucial to the strengthening of the focal adhesion [53]. What features of the vinculin tail and F-actin allow for this crucial interaction? Using a combination of experimental electron microscopy and computational protein docking methods Janssen et al. [36] have addressed the Vt interaction with F-actin. They suggest two patches of basic residues on the surface of Vt link with two patches of acidic residues on the surface of F-actin. One of the actin acidic patches is on the *n-2* subunit and the other is on the *n* subunit. Their study also suggests that full-length vinculin in its closed conformation would be unable to bind along the actin filament as D1 would clash with regions of the actin filament. It is unclear dynamically how D1 would inhibit the link to F-actin, which of the acidic patches on F-actin are more critical to linking Vt, and whether the suggested conformation of activated vinculin [42] would allow for Vt to link these acidic patches. These issues are addressed in the first section on the binding of vinculin along the actin filament.

It has also been suggested that interaction of Vt with F-actin can inhibit actin polymerization [54]. During cell movement actin filaments in the lamellipodia will polymerize at their barbed-ends [31]. The polymerization is involved in membrane protrusion at the leading edge of a migration cell [55]. One line of evidence supporting the notion that Vt can inhibit actin polymerization comes from studies of the bacterial effector IpaA [54], [56]. IpaA can stop polymerization of actin filaments of its target cell and can even cause depolymerization of the actin filaments. The direct effect of IpaA is to activate vinculin for capping of F-actin at the barbed-end. Although the mechanisms of vinculin activation for F-actin capping by IpaA are not clear, it is suggested from these studies that vinculin can cap the actin filament and prevent its polymerization. Further lines of evidence for vinculin capping of actin filaments come from other studies showing that Vt (isolated form other vinculin residues) can catalyze G-actin nucleation through interaction with the barbed-end of G-actin [57]. Most recently, Le Clainche et. al. [58] have explored capping of F-actin by vinculin *in vitro*. They used pyrenyl-labeled actin fluorescence [59], [60] to assay the polymerization of G-actin into F-actin before and after introduction of Vt *in vitro*. Introduction of Vt prevents polymerization of F-actin. Their results suggest that residues 1044–1066 of Vt are critical to capping of F-actin by vinculin. Several questions arise concerning this capping of actin filaments by vinculin that the second section of this study on the capping of F-actin will address: what is the structure of F-actin capped by Vt? What residues and surface regions of the barbed-end are critical to interaction with Vt? How favorable or stable are these interactions between the barbed-end of F-actin and Vt?

The interaction between vinculin and actin is of importance not only to efforts aimed at understanding focal adhesion formation via talin and vinculin, but also to efforts aimed at understanding the role of vinculin in regulating actin dynamics, or the role of vinculin in other cellular processes. A study by Wilins and Lin [61] established a role of vinculin in regulating actin dynamics, and more recently Huveneers *et al.* suggested vinculin to be involved in stabilizing force-dependent remodeling of endothelial cell-cell adhesions [62]. This study investigates both the interaction of vinculin along the actin filament and the capping interaction of vinculin with the barbed-end of F-actin. Molecular dynamics simulations are used to probe the interaction of vinculin along the actin filament using: (a) a structure of only Vt interacting with actin subunits *n* and *n-1*, (b) a structure of vinculin is its closed conformation, and (c) a structure of vinculin in its suggested activated conformation [42]. Furthermore, a similar molecular dynamics approach is used to determine the likely structure, dynamics, and energetics of the interaction between Vt and the capping end of F-actin. Finally, computational techniques are used to evaluate an additional conformational change in full-length vinculin (beyond the suggested activation of vinculin at D1) and explore the possibility of an interaction between the barbed-end of the actin filaments and vinculin in this second open structure.

## Results/Discussion

### Vinculin binding along the actin filament

#### Simulation of Vt binding along the actin filament

**Figure 2:**
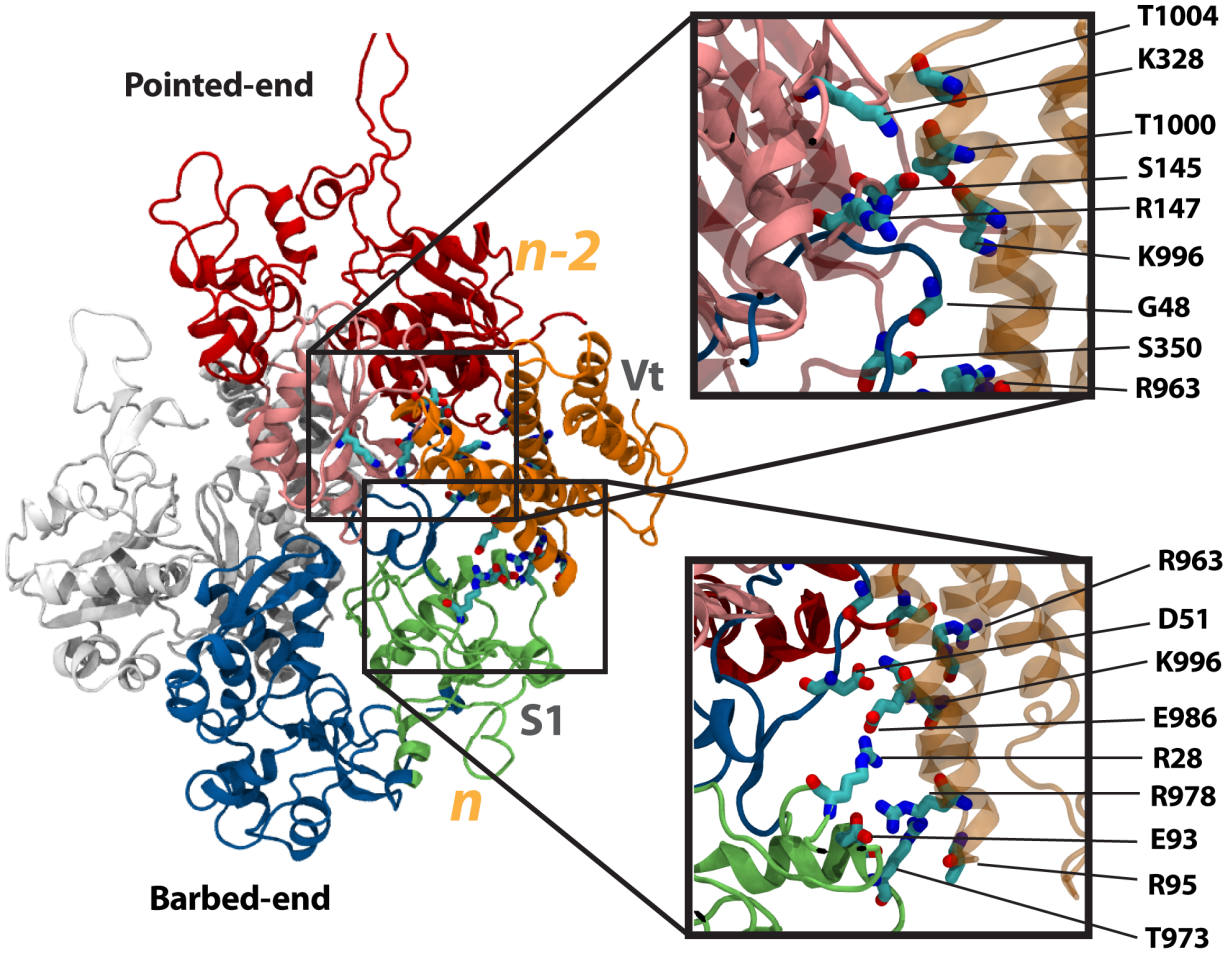
Interaction of Vt with actin along the filament. The interaction between Vt (orange) and the F-actin is simulated using molecular dynamics. Vt forms two sets of interactions with F-actin along the filament: (1) an interaction with S3 of *n-2* (top inset), (2) and interaction with S1 of *n* (bottom inset). Four interactions are formed between Vt and *n-2*: T1004 with K328, T1000 with R147, K996 with S145, and R963 with S350. Five interactions are formed between Vt and *n*: K996 with G48, E986 with R28, K996 with D51, T973 with R95, and R978 with E93. Actin subunit *n* is shown in blue, and subunit *n-2* is shown in red.

Linkage to actin filaments is important for formation of stable focal adhesion structures [53]. At the focal adhesion, the reinforcing agent vinculin binds talin through D1 and is suggested to bind actin through Vt. Molecular dynamics was used to simulate the interaction between Vt and the actin filament. A structure of the actin filament was used with 3 subunits, each in their F-actin conformation (as opposed to the G-actin conformation) [63]. Vt was initially oriented to approach F-actin for binding along the filament (Figure 1). Actin subunits *n* and *n-2* together form the filament surface that Vt would approach. Vt is initially placed more than 15 Å away from F-actin. A small nudging force was used briefly (less than 1 ns) to accelerate Vt towards the actin filament. Nudging Vt towards actin reduces the entropic barrier to an interaction between Vt and actin, and had been used previously for simulation of binding events [48], [51]. Following the brief nudge, the two-molecule system – with Vt and F-actin – was simulated with no external forces or constraints for 15 ns. A final complex was formed with Vt linked along the actin filament (Figure 2).

Two interfaces were formed between Vt and F-actin: an interface between Vt and actin subunit *n-2*, and an interface between Vt and actin subunit *n*. Vt interacted with the S3 subdomain of *n-2*. There are four interactions between polar and charged residues stabilizing this interaction (Figure 2): T1004 with K328, T1000 with R147, K996 with S145, and R963 with S350. K996 and R963 are basic residues on Vt and have previously been suggested to link with F-actin [36]. Vt also interacted with S1 of *n*. At this interface three salt-bridges are formed: E986 with R28, K996 with D51, and R978 with E93. There were an additional two interactions at the S1-Vt interface between polar and charged groups: K996 with G48, and T973 with R95 (Figure S1). Both the interactions with *n-2* and with *n* contribute to stabilizing the Vt-actin linkage. The existence of salt-bridges between *n* and Vt suggest this interface to be more dominant.

To compare the influence of the two interfaces on the stability of the Vt-F-actin complex, the potential energy of the interacting residues at both interfaces were calculated throughout both simulations. Molecular complexes will likely adopt conformations and arrangements that reside in local potential energy minima [64]. Of the two interfaces, whichever is contributing more towards reducing the potential energy of the system is likely more dominant in effecting the formation of the molecular complex. The calculations showed that the interactions between Vt and F-actin at the interface with S1 of *n* store 150 Kcal/mol of potential energy, considerably more than the interaction of Vt with *n-2* (Figure S2). This would be expected given the absence of salt-bridges at the Vt-*n-2* interface.

The two interfaces, one between Vt and *n* and the other between Vt and *n-2*, are comparable to the two acidic patches on the F-actin surface previously predicted to be involved in linking Vt to F-actin [36]. What was previously described as the upper Vt binding site on F-actin is the interface between Vt and *n-2* seen in our molecular dynamics simulations. And the previously described lower Vt binding site on F-actin is the interface between Vt and *n*. The molecular dynamics simulation of Vt interacting with F-actin confirms the predictions of Janssen et al [36]. Evaluation of the potential energy changes resulting from each set of interactions predicts that of the two sites, the lower binding site, or the interaction between Vt and S1 of *n* is more favorable and more important to linking F-actin to focal adhesions.

#### Simulation of closed vinculin binding along the actin filament

**Figure 3:**
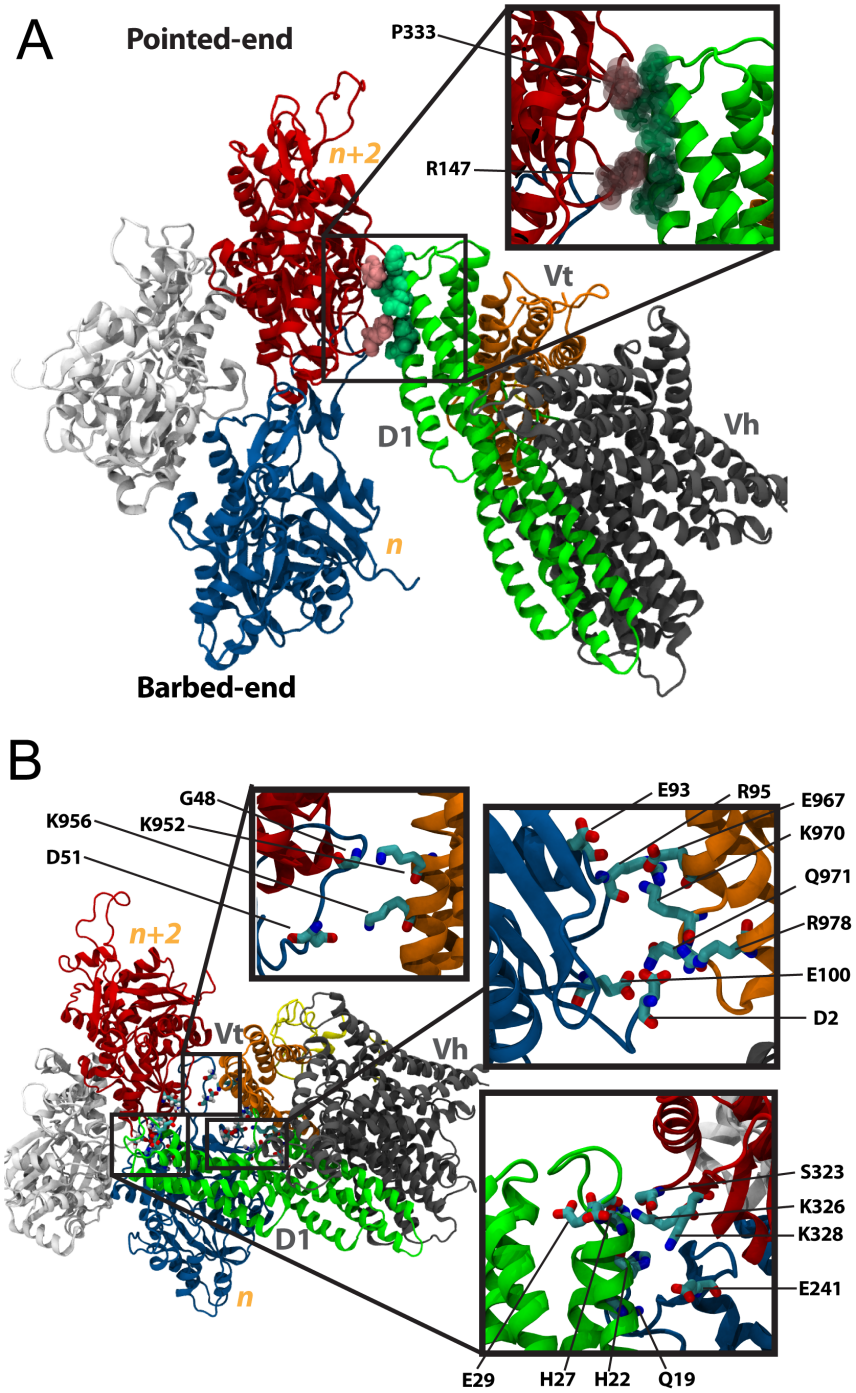
Interaction between vinculin in its closed and open conformation with F-actin along the filament. (A) The interaction between full-length vinculin in its closed conformation and F-actin is simulated with molecular dynamics. Initially vinculin is with the same orientation relative to F-actin that was used for simulation of Vt. After 15 ns of simulation no stabilizing interactions are formed between vinculin in the closed conformation and F-actin. The closed vinculin makes contact with P333 and R147 of F-actin, as shown in the inset. The contacts result in steric clashes and are followed by movement of vinculin in its closed conformation away from F-actin. (B) A structure of vinculin in its open conformation has previously been suggested [42]. Using this structure, the interaction between vinculin in its open conformation and F-actin is simulated. The vinculin molecule is initially oriented towards actin with the same orientation that was used for simulation of Vt. After 15 ns of simulation the open conformation of vinculin has formed three sets of binding interactions along the actin filament: (1) an interaction if Vt (orange) with S2 of subunit *n* (top left inset), (2) and interaction of Vt with S1 of subunit *n* (top right inset), and (3) and interaction of D1 (green) mainly with S3 of subunit *n-2* (bottom right inset).

Numerous studies have described the vinculin head domains as being auto-inhibitory and preventing the linkage between Vt and F-actin [37], [39]–[41], [65], [66]. The study by Janssen et al [36] also predicted that D1 of the vinculin head would stericly clash with F-actin and prevent linkage between Vt and F-actin. To evaluate the impact of D1 and other vinculin head domain residues on the interaction with F-actin, molecular dynamics simulations were run with full-length vinculin oriented towards the two Vt binding sites along the filament. Full-length vinculin was simulated with both its closed conformation and with the suggested open conformation [42]. The vinculin structures were initially placed 15 Å away from F-actin and accelerated towards the F-actin binding sites using a small force for less than 1 ns [51]. In simulation with both structures, after the initial nudge the system is simulated for 15 ns in the absence of any external forces or constraints. The simulation of vinculin in a closed conformation did not result in formation of a complex between vinculin and F-actin (Figure 3A) and the simulation of vinculin with the predicted open conformation resulted in formation of a complex between vinculin and F-actin (Figure 3B). The closed vinculin structure is included as a negative control and its failure to bind supports the binding results of the open conformation.

Two residues on the surface of F-actin along the filament made contact with the closed conformation of vinculin during the 15 ns simulation (Figure 3A): P333 and R147. These residues contact residues Q93, Q96, S97, A42, A45, and A44 of vinculin in the closed conformation. The contact was not favorable and vinculin moved away from F-actin following the contact (Figure S3). The failure of vinculin in the closed conformation to bind F-actin was expected [36]. The molecular dynamics simulations confirmed that a conformational change is necessary for binding along the F-actin filament and have demonstrated a trajectory of the steric repulsion between D1 and F-actin.

#### Simulation of open vinculin binding along the actin filament

In the simulation of open vinculin with the actin filament three interfaces were formed between vinculin and F-actin (Figure 3B). K956 linked with R51 and G48, and K952 linked with G48 at one interface – between vinculin domain Vt and S2 of *n*. E967 linked with R95, R978 linked with E93, K970 linked with E93, Q971 linked with E100 and R978 linked with D2 at the second interface – between vinculin domain Vt and S1 of *n*. And interestingly, H27, E29, and E28 of vinculin domain D1 linked with S323, K326, and K328 of *n-2* (subdomain S3). This third interface is unique to the simulation with an open vinculin conformation. The first and second interface are similar in location to the lower binding site previously described for interaction of Vt (isolated from the vinculin head domains) with F-actin. No interface was formed between Vt and the upper binding site on F-actin. The absence of an interface with the upper binding site is consistent with our prediction that the lower binding site, at S1 of *n*, is more critical to an interaction between F-actin and vinculin. All interactions persisted throughout most of the 15 ns simulation (Figure S4)

It remains possible that stochastically, if further simulations between open vinculin and F-actin were performed an interaction between vinculin and the upper binding site would present itself. This simulation points out the consistency of an S1-Vt interface. The third interface that linked vinculin to F-actin was formed between D1 and Vt. Previous studies had not considered a possible interaction between Vt and F-actin. In the closed conformation, the charged residues of D1 that interact with F-actin – E29 and E28 – are linking D1 to Vt. Previous studies of vinculin in its closed conformation binding to F-actin have failed to recognize any interaction between these acidic residues and their basic counterparts on *n-2*. The binding interface is near R1008 and is not accessible in the closed vinculin conformation. In the open conformation however, D1 has separated from Vt, and E28 and E29 are available for linking to basic residues on the F-actin surface. Our simulations demonstrated that the well-characterized interaction between Vt and F-actin would bring these residues in close proximity to *n-2* allowing them to further link vinculin to F-actin. The suggested open conformation of vinculin facilitates the binding of vinculin along the actin filament not only by removing the steric hindrance to a Vt-F-actin interaction, as previously suggested [36], but also through contributing an additional binding interface between D1 and F-actin.

Comparing the potential energy stored in the interaction between D1 and Vt with the potential energy stored in the Vt-F-actin interface (Figure S5), it is clear that as with binding of Vt along the actin filament, the interaction with S1 is more favorable. The D1 interaction does contribute to reducing the potential energy of the system but not to the same level as Vt. Considering the mainly ionic nature of the interactions between Vt and S1 of *n*, it is not surprising that the Vt-S1 interaction is favorable.

#### Discussion of vinculin binding along the actin filament

Comparing the three simulations of vinculin binding along the F-actin filament (Figure S6) it is evident that Vt does interact favorably with F-actin when isolated from D1 of the vinculin head domain. The suggested conformational change leading to open vinculin [42] is evaluated in these simulations. Only after the movement of D1 away from Vt did vinculin interact favorably with F-actin, and no further conformational changes proved necessary to allow the F-actin interaction. Elsewhere it has been suggested that complete separation of Vt from all vinculin-head domains is necessary to allow for binding of vinculin along the actin filament [33]. Our results contend that D1 movement away from Vt is necessary and sufficient for vinculin activation.

The suggested open vinculin conformation resulted from previous simulations that considered the transient interaction of vinculin with nearby actin filaments while linked to talin [42]. Considering that the S1 binding site on subunit *n* of actin was shown to be the site of primary linkage between Vt and S1, it remains possible that a transient interaction between these two regions coupled with stress transduced to vinculin through its linkage to talin could catalyze the movement of D1 away from Vt. Alternatively, the clash between D1 and F-actin, if coupled with stress transduced to vinculin through its linkage to talin could also catalyze the movement of D1 from Vt. Exploring those possibilities, and exploring the trajectory of vinculin activation through a simultaneous interaction with actin and talin – suggested both by our previous simulations [42] and previous experimental results [40] – would make a valuable future study. The results presented here confirm the D1 movement facilitates F-actin binding, but beg the question of how that D1 movement could be achieved *in vivo*.

Understanding the binding of vinculin along the actin filament is important because of the role of actin filaments at focal adhesions [53]. The actin filament network provides the mechanical stability needed by the cell. At focal adhesions, this network is coupled to the ECM and the external environment. Any forces originated from within the cell or from without the cell can only be mechanically reinforced by the actin network if this coupling is complete. In the motile cell, the actin network forms short parallel actin fibers near to the cell edge [52]. It is to these shorter parallel actin fibers that the newly forming nascent adhesions aim to connect with. Our simulations predict that the vinculin from the maturing nascent adhesion would facilitate the F-actin linkage at the stage of maturation in which mechanical stresses are present. If at the earliest stages of nascent adhesion formation, mechanical stress in the amount necessary to separate D1 from Vt is absent, then the linkage to Vt is likely through other molecules, such as talin itself. As the nascent adhesion matures, and the stress around the maturing nascent adhesion intensifies vinculin becomes activated and linkage to the actin filaments intensify. In this way vinculin can be described as a reinforcing agent.

One possible source of stress that could activate vinculin at the nascent adhesion would be the retrograde flow of the actin fibers within the lamellipodium [67]. The moving actin filaments would brush against focal adhesion proteins. Vinculin in its closed conformation, linked to talin at D1, could collide with the flowing actin filaments, and the collision could bias the population of vinculin to bring about a population-shift from closed vinculin to open vinculin [68]. Further evidence supporting the notion that actin flow at the lamella and the lamellipodium can stress the focal adhesion molecules come from a recent study describing the formation of an actin arc structure [69]. At the leading edge of the migrating cell the actin filaments show polymerization followed by retraction. The polymerizing actin filament will cause protrusion of the cell membrane. The retraction and rearward movement of the actin filaments correlates with retraction of the cell membrane. Interestingly, the actin filaments form arc like structures around adhesions complexes during the retraction stage of the cycle.

During retrograde flow of the actin filaments, at times the focal adhesion or nascent adhesion linked to the actin filament will undergo slippage [70]. One possibility is that although the integrin and other focal adhesion molecules would disconnect from the moving actin filament, vinculin would remain connected. After the movement, the vinculin would reconnect to another adhesion structure preventing further flow of the actin filament. In such a way the focal adhesion proteins would act a molecular clutch providing a variable contact between F-actin and integrin [70]. It is possible the stress on vinculin during the slippage event would surpass the energy barrier needed to activate vinculin and cause further vinculin conformational changes. What role would any second vinculin conformational change play either at the focal adhesion or in regulating the dynamics of actin filaments? One possibility is the second conformational change could regulate the capping of actin filaments by vinculin.

### Vt capping of the F-actin barbed-end

Using pyrenyl-labeled actin to assay F-actin polymerization Le Clainche et al [58] demonstrated *in vitro* that Vt can effectively cap the barbed-end of actin. Capping of the actin filaments by vinculin has been demonstrated in cells affected by IpaA [71]. In such cells the capping of F-actin serves to depolymerize the actin filaments and make the cells compliant for Shigella invasion [54]. It is unclear if capping of actin filaments could play a role at sites of focal adhesions. It is also unclear if vinculin at focal adhesions is able to cap the actin filaments. A step towards clarifying both possibilities is to understand the nature of F-actin capping by vinculin.

**Figure 4:**
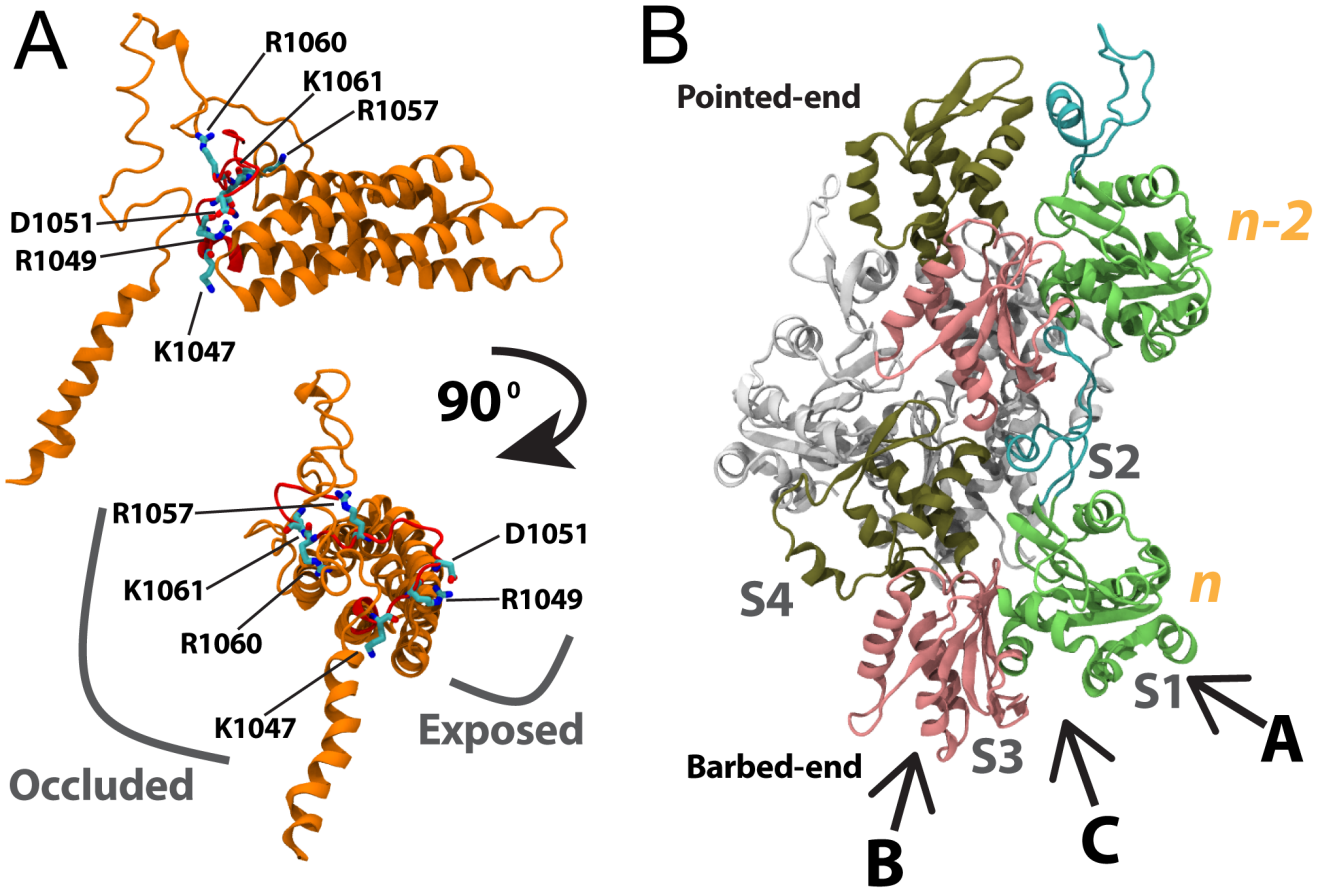
Basic C-terminus residues on the vinculin tail and the barbed-end of actin. (A) Recent experimental evidence suggests Vt can bind the barbed-end of an actin filament [58]. Residues 1044 to 1066 (red) of the C-terminus region of Vt are likely involved in capping of the actin filament. There are several charged residues within the C-terminus region: K1047, R1049, D1051, R1057, R1060, and K1061. These residues would interact with the barbed end of actin either through the surface of Vt that is exposed to solvent and F-actin, or through the surface of Vt that is occluded by the vinculin head domains. The top view of Vt is rotated by 90 degrees to produce the bottom view of Vt. The interaction of Vt with the barbed-end of actin is simulated with either the exposed surface of Vt oriented towards actin, or the occluded surface of Vt oriented towards actin. (B) Vinculin could approach the barbed-end of actin for capping from three directions: (A) approaching S1 of the *n* subunit, (B) approaching S3 of the *n* subunit, or (C) approaching S1 and S3 of the *n* subunit. Binding to actin from any of these directions would result in capping of the barbed-end of actin and prevent further polymerization of actin at the barbed-end. S1 is shown in green, S2 in cyan, S3 in pink, and S4 in tan.

The vinculin tail residues implicated in F-actin capping reside in the C-terminus region and have also been implicated in interaction with the lipid membrane [72]. The last 21 residues of vinculin consist of a number of charged and basic residues that are predicted to readily interact with acidic residues at the actin barbed-end or on acidic phospholipids (Figure 4A). In its closed conformation vinculin head domains occlude access to most of these residues. With Vt isolated from the vinculin head domains the last 21 residues could interact with the barbed-end of F-actin either through the surface of Vt that would be occluded by vinculin head domains, the occluded surface, or through the surface of Vt already exposed to solvent, the exposed surface of Vt. From the structure of Vt it is predicted that the occluded face of Vt would better link the barbed-end given the higher density of charged residues at this surface (Figure 4A).

Newly added actin subunits would interact with the barbed-end of F-actin. Examination of the F-actin structure predicts that the D-loop of subunit *n* interacts favorably with the interface between S1 and S3 of subunit *n-2* (Figure 1). Specifically, residues 283–294, 139, 140, 143, 346, 351, and 374 of the barbed-end are implicating in stabilizing additional actin subunits by interacting with their D-loop structures [28]. Recent high resolution imaging of the actin filament pointed-end confirms the likely interaction between the D-loop of a newly polymerized actin monomer and the interface between S1 and S3 at the barbed-end of the actin filament [32].

Capping of F-actin by CapZ and other capping proteins prevents actin polymerization by occluding access to S1 or S3 [32]. Capping of F-actin by Vt would then likely result from interaction of Vt with S1 of the barbed-end, S3 of the barbed-end, or both subunits S1 and S3 of the barbed-end (Figure 4B). The interaction of Vt with F-actin is evaluated using molecular dynamics. Vt is simulated initially oriented towards the barbed-end towards either (A) S1 only, (B) S3 only, or (C) towards S1 and S3. Each orientation is simulated both with the exposed face of Vt initially oriented towards the barbed-end and with the occluded face of Vt initially oriented towards F-actin (Table S1).

#### (A) Binding of Vt to S1 of the actin barbed-end

**Figure 5:**
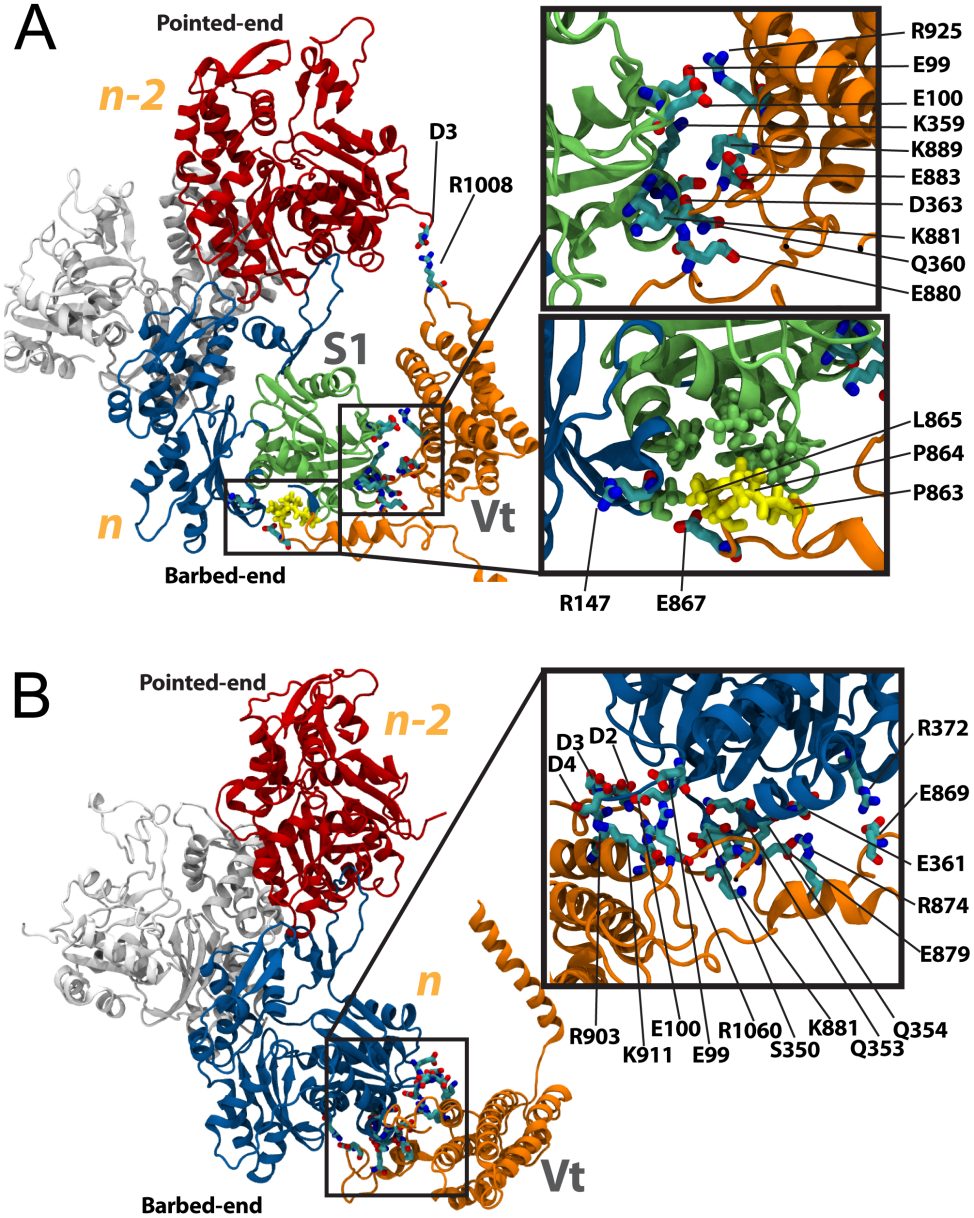
Capping of the actin filament at S1. (A) Vt (orange) is simulated interacting with the barbed-end of actin. The Exposed surface of Vt is initially oriented towards actin, and Vt is arranged to approach S1 of the barbed-end. After 15 ns of simulation two binding surfaces are produced: (i) electrostatic linkage between charged Vt residues and S1 (top inset), and (ii) hydrophobic insertion of 3 Vt residues into a hydrophobic patch between S1 and S3. (B) Vt is simulated interacting with the barbed-end of actin with its occluded surface initially oriented towards actin. After 15 ns of simulation a binding surface is produced between Vt and S1 of the barbed-end (inset). Several interactions are formed at this interface: R903 interacts with D2 and D3 of S1, K911 with D4, R1060 with E99 and E100, K881 with S350, E879 with Q354, R874 with E361, and E869 with R372. Subunit *n* is shown in blue, and subunit *n-2* is shown in red.

Simulation of Vt interacting with S1 of the barbed-end (Figure 5) showed formation of a significant link both between the exposed surface of Vt and S1, and between the occluded surface of Vt and S1. The link between the exposed surface and S1 involves the hydrophobic insertion of P863, P864, and L865 into the small hydrophobic patch between S1 and S3 (Figure 5A). Additionally, R925 links with E99 and E100, K889 links with D363, E883 links with K359, E880 and E884 link with D882 and Q360, and K881 links with S358. Six salt-bridges and a few polar interactions dominate this interaction and stabilize the linkage between the exposed surface of Vt and S1 (Figure S7). The link between the occluded surface and S1 involves the following stable interactions (Figure 5B and S8): R903 with D2 and D3, K911 with D4, R1060 with E99 and E100, K881 with S350, E879 with Q354, R874 with E361, and E869 with R372. These ionic and polar links are comparable to the links between the exposed face of vinculin and S1. Comparison of the potential energy changes resulting from both interactions (Figure S9) confirms that both are favorable, storing as much as 350 Kcal/mol of potential energy, and are equally likely to occur.

The hydrophobic insertion into the groove between S1 and S3 by Vt is consistent with previous studies examining the structure of CapZ bound to the barbed-end of F-actin [32]. CapZ hydrophobic residues insert in the same hydrophobic patch between S1 and S3. Basic residues on CapZ also interact with acidic residues at the barbed-end. A study of β-thymosin/WH2 capping actin filaments [73] also confirms the role of a hydrophobic insertion between S1 and S3 in capping actin filaments. Our simulations suggest the hydrophobic insertion also plays a role in stabilizing F-actin capping by Vt. Although P863, P864, and L865 were able to bind the barbed-end in simulation with the exposed surface of Vt, these residues lie within the flexible loop region. Whether in a full-length vinculin structure these loop region residues would be available for F-actin capping is unclear.

#### (B) Binding of Vt to S3 of the actin barbed-end

**Figure 6:**
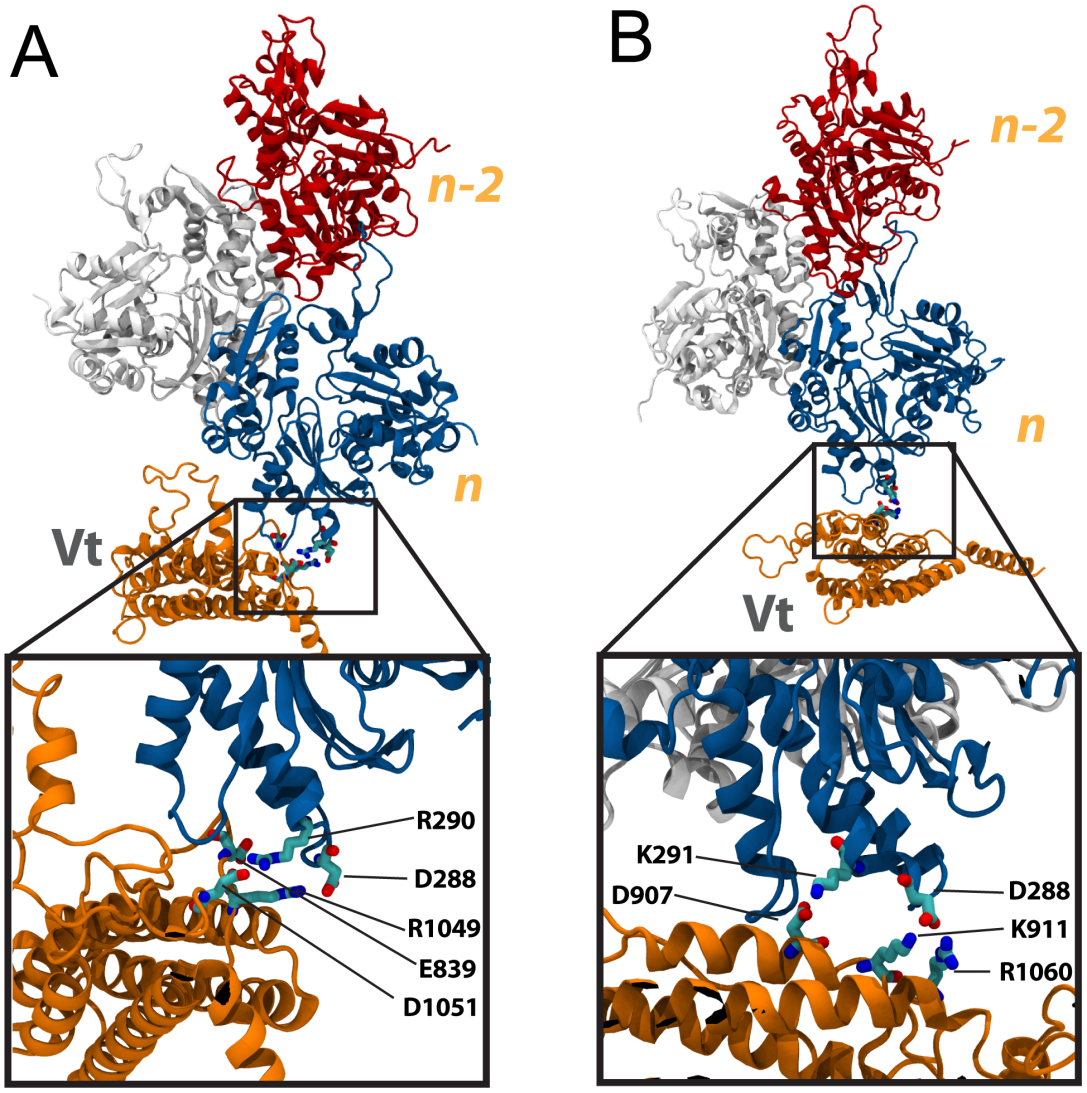
Capping of the actin filament at S3. (A) Vt (orange) is simulated with its exposed surface initially oriented towards the S3 subdomain of the barbed-end of F-actin. After 15 ns a small interacting interface is formed (inset). There are three interactions at this interface: R1049 with D288, D1051 with R290, and E839 with R290. (B) Vt (orange) is simulated with its occluded surface initially oriented towards S3 of the *n* subunit. After 15 ns of simulation two interactions are formed between the occluded surface of Vt and S3 of the barbed-end (inset): R1060 and K911 interact with D288, and D907 interacts with K291. Subunit *n* is shown in blue, and subunit *n-2* is shown in red.

Simulation of Vt interacting with S3 of the barbed-end (Figure 6) showed little linkage being formed between Vt and S3. The exposed surface of Vt formed three salt-bridges between Vt and F-actin (Figure 6A and S10): R1049 with D288, D1051 with R290, and E839 with R290. The occluded surface of Vt formed two salt-bridges between Vt and F-actin (Figure 6B and S11): R1060 and K911 with D288, and D907 with K291. Although more links were formed at the exposed surface of Vt, calculation of the potential energy stored in the interactions shows interaction with the occluded surface of Vt is more favorable (Figure S12). Both sets of salt-bridges have at least 150 Kcal/mol of potential energy stored. Because basic residues of the occluded surface of Vt were able to get closer to the acidic residues of S3, these ionic links were calculated to store a maximum of over 250 Kcal/mol. In comparison to interactions with S1, the interactions with S3 are less profound and likely play less of a role in the capping of F-actin by Vt.

#### (C) Binding of Vt to S1 and S3 of the actin barbed-end

**Figure 7:**
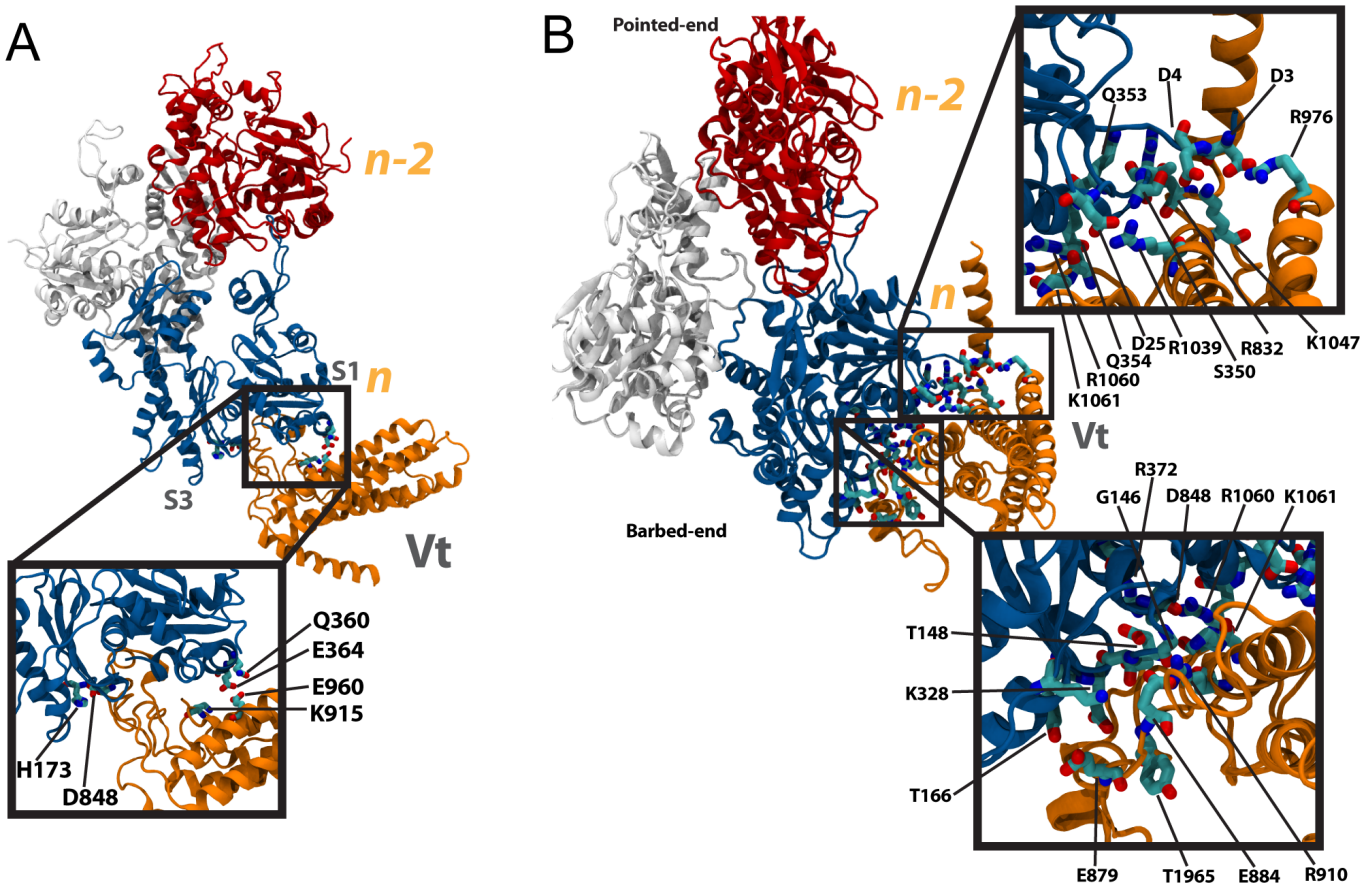
Capping of the actin filament at S1 and S3. (A) Vt (orange) with its exposed surface initially oriented towards both S1 and S3 is simulated interacting with the barbed-end of F-actin. After 15 ns of simulation a binding interface is formed between the exposed surface of Vt and the barbed-end (inset). D848 of Vt interacts with H173 of S3, while two interactions are formed between Vt and S1: K915 with E364, and E960 with Q360. (B) Vt (orange) is simulated with its occluded surface initially oriented towards both S1 and S3. After 15 ns of simulation the occluded surface of Vt forms a large interface at the barbed-end of Vt, interacting with both S1 and S3. Both insets together show the interactions at this large interface. Subunit *n* is shown in blue, and subunit *n-2* is shown in red.

The third set of capping simulations oriented Vt towards both S1 and S3 of the barbed end. In simulation with the exposed surface of Vt only 1 stable salt-bridge was formed with F-actin after 15 ns of simulation (Figure 7A and S13): K915 with E364. Two weaker polar interactions were also formed: D848 with H173, and E960 with Q360. The polar interactions likely contribute less to stabilizing the complex. In contrast to capping with the exposed surface, F-actin capping with the occluded surface of Vt was shown to be very stable and likely favorable (Figure S14). A large interface was formed between the occluded surface of Vt and the barbed end of F-actin (Figure 7B). Numerous close links were formed: E884 with K328 and R147, D848 with R372, R910 with G146, E879 with T166, T1065 with E167, K1061 with T143 and T148, R1039 with D25, R1060 with Q354, R832 with Q353 and T351, K1047 with D4, R976 with D3, and K1047 with S350. Calculation of the potential energy stored in this large interface confirmed that it is indeed favorable (Figure S15). A total of more than 550 Kcal/mol of potential energy was stored in the large interface between the occluded surface of Vt and F-actin. The interaction between the exposed surface and F-actin only stored 100 Kcal/mol. These results suggest that the occluded surface of Vt is more likely the surface involved in capping of F-actin.

#### Discussion of Vt capping of the actin barbed-end simulations

A total of six simulations were run to explore the possible interaction between Vt and the barbed-end of F-actin (Figure S16). Of these simulations, one resulted in the likely structure of Vt capping an actin filament (Figure 7B): the occluded surface of Vt bound to S1 and S3. This interaction contained the largest number of ionic and polar links between Vt and the barbed-end, and the highest level of potential energy stored within the linkage. Overall, interactions with S1 were shown to be more favorable than interactions with S3, and interaction with the occluded surface of Vt was shown to be more favorable than interaction with the exposed surface of Vt. Interaction with S3 and interaction with the exposed surface did show favorable linkages, but less favorable than interaction with S1 or interaction at the occluded surface.

S1 of subunit *n* was shown to be significant both to the binding of vinculin along the actin filament and to the capping of the actin filament. The feature of S1 that lends it such a significant role is the prevalence of acidic residues on its surface. The existence of the acidic residues have previously been recognized elsewhere both for binding along the actin filament [36] and for capping of the actin filament [28], [74]. These acidic residues play a role not only in vinculin binding but also in binding to other proteins [75].

The results from simulations of F-actin capping are consistent with the understanding of F-actin capping in general [74]. Two interactions stabilize the subunits that make up an actin filament: (1) hydrophobic interaction between the D-loop of an *n* subunit and the hydrophobic patch between S1 and S3 of an *n-2* subunit, and (2) interactions between acidic residues on S1 and S3 on *n-2* and basic residues on *n* [28]. In one of the simulations, three hydrophobic residues, P863, P864, and L865, inserted into the hydrophobic patch between S1 and S3. Although these residues are from the loop region and might not be involved with F-actin capping, the interaction demonstrates dynamically the importance of the hydrophobic interaction. The strongest link with the barbed-end of F-actin was formed when interacting with both S1 and S3, consistent with previous notions [76]. In order for an interaction to prevent polymerization the two key mechanisms for stabilizing the new actin subunit should be disrupted. Binding of Vt to the barbed end can disrupt both mechanisms. Vt can be an effective F-actin capping protein.

The capping of F-actin by vinculin has been demonstrated in cells affected by Shigella and its effector IpaA [71]. Can vinculin cap F-actin at focal adhesion, and can this capping play a role in regulating actin dynamics at focal adhesions? The answer is unclear. A hypothesis would be that indeed it could. Considering the role actin network movement and dynamics can play in regulating focal adhesion growth [77], its possible that as a negative feedback loop vinculin could regulate the dynamics of those actin networks. Vinculin would then be acting not as a binary switch, shifting between a closed and an open conformation, but as a variable switch, shifting between a closed, an open I, and an open II conformation. The open I conformation would correspond to the structure of vinculin with D1 moved away from Vt allowing for linking of F-actin to the focal adhesion. The open II conformation would involve a second conformational change that would expose the residues predicted in these simulations to be critical for linking Vt to both S1 and S3 of the F-actin barbed-end.

### The vinculin open II conformation

An additional conformational change is necessary for vinculin to be able to cap actin filaments. The simulations of Vt capping of actin filaments suggest that the occluded surface of Vt forms the most likely interaction with the interface between S1 and S3. The occluded surface is normally in contact with D4 residues. Movement of D1 away from Vt, which was shown to be sufficient for allowing vinculin to bind along actin filaments, does not result in dissociation of Vt from D4. The occluded surface of Vt, critical to F-actin capping, requires additional conformational changes in vinculin beyond D1 movement. Le-Clainche et al [58], [78] also suggest a second vinculin conformational change is necessary to allow for vinculin capping of the actin-filaments.

The interface between D4 and Vt has been implicated elsewhere as critical to Vt activation in general. Chen et al [78] describe a pincer-like mechanism to vinculin activation in which both the interface of D1 with Vt and the interface of D4 with Vt is disrupted to allow Vt to leave the pocket formed by the vinculin head domains and link with F-acitin. In another study, Cohen et al [65] demonstrate that both interactions between Vt and D1 and interactions between Vt and D4 are critical to maintaining an auto-inhibited conformation. The simulations in this study suggests that disruption of the key interactions between Vt and D1 coupled with movement of D1 away from Vt is sufficient to allow binding of vinculin along the actin filament. The interaction between Vt and D4 could however play a critical role in regulating Vt capping of actin filaments.

The separation of D1 from Vt was simulated by assuming a cooperative activation mechanism and introducing stretch of vinculin to be consistent with that mechanism [42]. The source for D4 separation from Vt is less clear. If Vt separates from D4 at the focal adhesions then perhaps the movement of the actin filaments across the developing focal adhesion can supply induce separation of Vt from D4. If however Vt separation from D4 is particular to cells affected by Shigella [71], then D4 separation would result from the interaction with IpaA. Whatever the source allowing fro D4 separation, it is likely that D4 would separate after D1 separation. In either scenario, the magnitude of force that would be needed to induce a second conformational change is telling of how likely it is that a conformation shift would occur. Estimating the *in vivo* magnitude of force accurately is not possible with molecular dynamics. The time-scale of computationally feasible molecular dynamics simulations is orders of magnitude faster than the *in vivo* time-scale. Nevertheless, the conformational changes we suggest here can be informative.

#### Molecular dynamics simulation of vinculin forming an open II conformation

**Figure 8:**
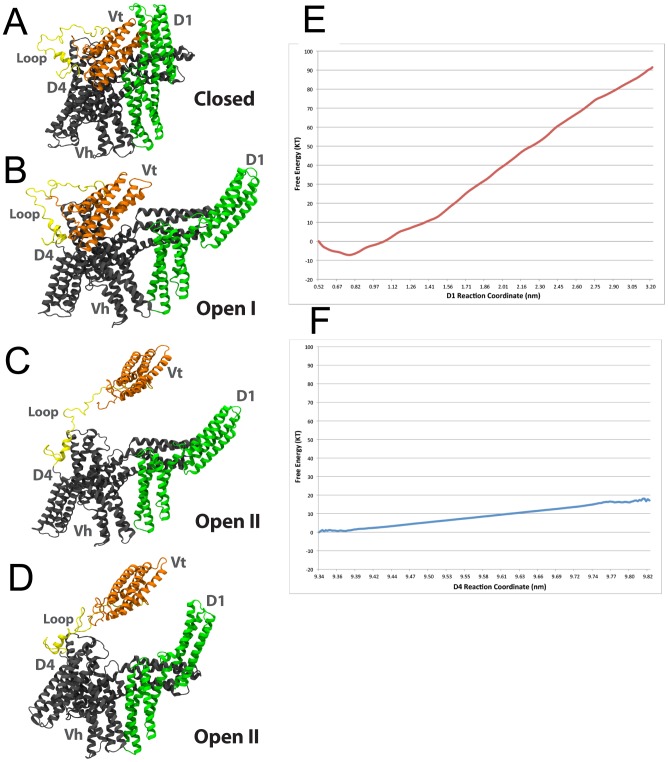
Formation of a vinculin open II conformation. In its closed conformation (A) vinculin is not likely to bind actin filament. Separation of D1 from Vt constitutes formation of the open I conformation (B) and molecular dynamics simulations suggest the open I conformation can bind along the actin filament. Another critical link between Vt and the vinculin head domains exists at the interface between D4 and Vt. Separation of Vt from D4 results in formation of an open 2 conformation (C). (D) When the open II conformation is equilibrated after being formed D1 moves towards Vt and the loop region reduces its strain. However, Vt remains detached from the vinculin head and D1 remains removed from Vt. In this open II conformation vinculin could interact with F-actin both along the filament and at its barbed-end. Using the umbrella sampling method [79] the potential of mean force for two reaction coordinates were calculated: (E) the distance between D1 and Vt, and (F) the distance between D4 and Vt. Progress along reaction coordinate (E) leads to formation of the open I conformation. Progress along reaction coordinate (F) leads to formation of the open II conformation. Formation of the open I conformation has a free energy barrier of 90 KT and the open I conformation is not likely to stay open without persistent energy input. The formation of the open II conformation following formation of the open I conformation has a smaller free energy barrier of less than 20 KT. The plateau at the end of reaction coordinate (F) suggests the open II conformation could remain open without external force.

In this section of the study, D4 is separated from Vt following separation of D1 from Vt. Vt is pulled at a constant velocity away from D4 and the simulation results in formation of a second open conformation, the open II (Figure 8). Following the separation the new structure is simulated without any external constraints or forces and allowed to relax to its new equilibrium structure (Figure 8). In the equilibrated open II conformation Vt has completely separated itself from the vinculin head domains. D1 is under less strain than in the open 1 conformation (Figure 8). Even with D1 moving towards Vt, Vt remains likely to bind along the actin filament in the open II conformation.

Formation of the open II conformation involves disruption of the interface between Vt and D4 (Figure S17). Five salt-bridges cooperate to maintain the Vt-D4 interface: R1057 with D841, R1060 with D856, R978 with K1047, K975 with E775, and R976 with E 770. Once all of these interactions are broken, then Vt readily moves away from the vinculin head domains and the linker residues begin to stretch. The dissociation of the salt-bridges is shown by calculation of the loss in potential energy stored in the D4-Vt interface (Figure S18). Although other interfaces between Vt and the vinculin head domains exist, these residues along with the residues at the D1-Vt interface are the crucial interactions after which Vt can completely separate from the vinculin head domains. The importance of the Vt-D4 interactions is consistent with previous studies [65], [78].

#### Umbrella sampling of the two vinculin activation conformational changes

To compare the likelihood of a open II conformation being formed following formation of the open I conformation, the potential of mean force [79] and free energy difference between the closed vinculin conformation, the open I conformation, and the open II conformation are calculated (Figure 8). About 90 KT of free energy must be supplied to vinculin for D1 to separate from Vt, and an additional 10 KT of free energy must be supplied to separate D4 from Vt. If 90 KT of free energy has been supplied by one of the possible sources – either cooperative binding between Vt and F-actin, or mechanical forces from movement of actin filaments near the focal adhesions – then it is possible that an additional 10 KT of energy would completely remove Vt from the vinculin head. As neither the open I conformation nor the open II conformation of vinculin are stable (Figure 8), the activation of vinculin would require exposure of vinculin to source of mechanical stress. The level of mechanical stress, less than 90 KT or greater than 100 KT, would determine which of the two open conformations are adopted.

That vinculin can undergo two conformational changes, and that which change takes place can be regulated by how much energy is supplied to vinculin is consistent with a role for vinculin as a molecular clutch [70]. At the focal adhesion, first a nascent adhesion would begin to form. As the mechanical stress on the nascent adhesion intensifies the talin molecules would begin to become activated for interaction with vinculin [80], and once vinculin links with talin, further mechanical stress of movement of actin filaments could cooperate to cause vinculin to change from its closed to the open I conformation. With the open I conformation vinculin could completely bind F-actin and talin. If at this stage, where the nascent adhesion has matured to a focal complex and is becoming a mature focal adhesion [81], the mechanical stress is even larger or the movement of F-actin is even stronger, then the additional stress could cause vinculin to transform from its open I conformation to an open II conformation and maintain a dynamic link between the moving actin filaments and F-actin. By such a mechanism the open II conformation would not only be significant for potentially allowing for vinculin capping, but also for allowing a dynamic link between F-actin and the focal adhesion. Complete separation of Vt from the vinculin head domains allows for Vt to link moving F-actin more easily.

It is known that the open II conformation, induced by IpaA, can cap actin filaments [56]. However, if open II is achieved at focal adhesion in cells not affected by Shigella, then an interesting question arises: are actin filaments capped at focal adhesions? If it is shown that an open II conformation will readily link the capping end of an actin filament (as predicted) then capping of actin filaments at highly stressed focal adhesions becomes likely.

#### Capping of actin barbed-end by the vinculin open II conformation

**Figure 9:**
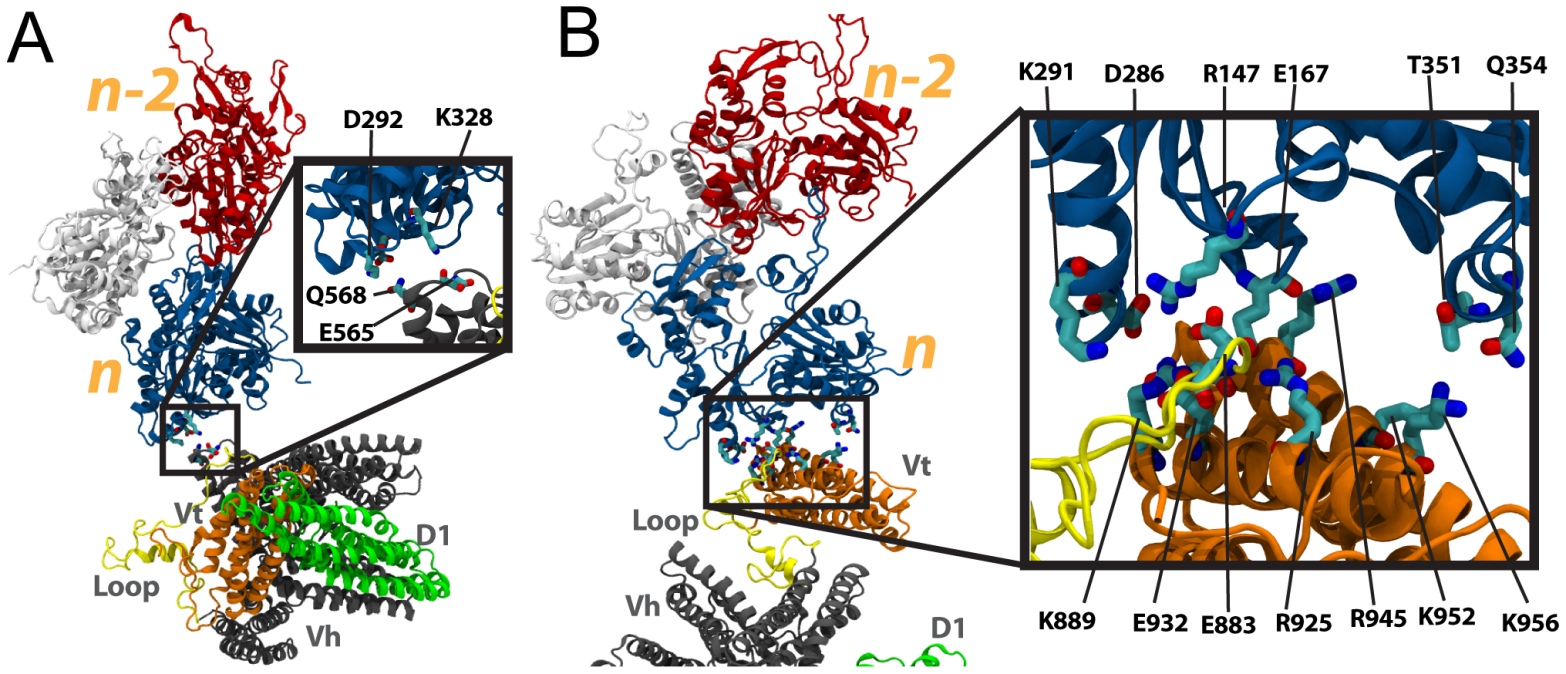
Capping of F-actin by vinculin in its closed conformation and open II conformation. (A) The surface of Vt (orange) likely to cap F-actin is normally occluded by vinculin head domains (black). D1 of the vinculin head is shown in green and the flexible loop region is shown in yellow. Subunit *n* of actin is shown in blue, and subunit *n-2* of actin is shown in red. Simulation with full-length vinculin in its closed conformation oriented towards S1 and S3 and with the same orientation as used for binding of the occluded surface of Vt to S1 and S3 resulted in formation of a small interface between Vt and the barbed-end of actin (inset). (B) The occlusion of Vt from F-actin can be removed after a potential conformational change. After 15 ns of simulation with this full-length vinculin structure in an open II conformation an interface has formed between Vt and F-actin (inset). Several interactions stabilize this interface: E932 with K291, R935 and K889 with D288, N943 with D286, E883 with R147, R925 with E167, K956 with T351, K952 with Q354, and R945 with Q349.

To evaluate if the open II conformation will bind the barbed-end of F-actin two simulations were produced, one with full-length vinculin in a closed conformation, and one with vinculin in the open II conformation (Figure 9). Simulation with the closed conformation showed minimal interaction with F-actin (Figure 9A). Initially Vt is attracted towards the barbed-end but residues in the vinculin head interacted with the barbed-end and vinculin moved away from F-actin (Figure S19). The brief interaction between the vinculin head and the barbed-end involved: E565 with K328, and Q568with D292 and K291. The simulation of vinculin in its closed conformation interacting with the barbed-end of actin is included as a negative control, and as expected, vinculin cannot bind the barbed-end without a conformational change. In contrast, simulation of vinculin in the open II conformation with F-actin resulted in the formation of an interface with many ionic and polar interactions stabilizing the open II vinculin at the barbed-end of actin filaments (Figure 9B): E932 with K291, R935 and K889 with D288, N943 with D286, E883 with R147, R925 with E167, K956 with T351, K952 with Q354, and R945 with Q349. Comparing the potential energy stored in the interface between vinculin and F-actin with the two structures confirms the favorability of the open II conformation for F-actin capping (Figure S20). Interaction of vinculin with the open II conformation with F-actin resulted in reduction of the potential energy of the complex by nearly 300 Kcal/mol. It is likely then that an open II conformation would result in F-actin capping, even at focal adhesion not affected by Shigella.

What role could F-actin capping play at focal adhesions? The open II conformation would form at the focal adhesion when stress levels are high. Perhaps, binding of vinculin even to the barbed-end of F-actin at these highly stressed focal adhesion would allow an additional layer of reinforcement. Alternatively, actin polymerization should occur at the leading edge of the moving cell. If the barbed-end is near to a stressed and maturing focal adhesion, polymerization of the actin filament near the focal adhesion would be unnecessary and the capping of F-actin at the focal adhesions would serve to limit actin polymerization only to the barbed end. The results from these simulation show that vinculin in an open II conformation can cap F-actin. Future experimental investigation is required to validate that this capping could occur at the maturing focal adhesion.

### Conclusion

The interaction between vinculin and actin was explored in this paper using molecular dynamics simulations in three sections: first, the interaction of vinculin along the actin filament was investigated, then, the interaction of Vt with the barbed-end of the actin filament, and finally, the possible interaction between an open II vinculin conformation and the actin filament. Simulation of the interaction along the actin filament confirmed that although Vt can bind along the actin filament, full-length vinculin in its closed conformation is inhibited from binding along the filament. The open I conformation, previously suggested as a conformation of activated vinculin, was able to bind along the actin filament as Vt had, confirming that it is likely the structure of activated vinculin. Simulation of Vt interacting with the barbed-end of F-actin confirmed that Vt could indeed prevent polymerization of the actin filament. Vt binds to the barbed-end of F-actin similar to other capping proteins [74] and can prevent the association of a new actin subunit with S1 and S3. Simulation of vinculin conformational changes beyond D1 separation and formation of the open I conformation revealed the possibility of an open II conformation. With the open II conformation vinculin could cap actin filaments, even at the focal adhesion.

In general, evaluating binding modes between a large filament such as actin and a large protein such as vinculin is a computationally demanding endeavor. In addition to the computational challenge posed by the size of the proteins, the simulation time needed to allow for a binding event to occur can be prohibitive. In the context of these computational challenges, this study has simulated the binding events with the following strategy: (1) reduce the size of the binding proteins by limiting the number of actin monomers to include in the simulations, (2) reduce the simulation time by including an initial nudge of vinculin towards actin, and (3) reduce simulation time by placing vinculin within 15 Å of actin. This has allowed for simulation of binding, but the study is limited by not having repeat binding events to capture the true scholastic nature of the binding.

An interesting scenario arises when considering the linkage of vinculin to actin at focal adhesions: vinculin is also linking to talin at focal adhesions, and so how are the three to be relatively oriented? In what order would the two binding events occur? The results from this study are insufficient to answer those questions accurately and additional simulations would be required to predict the binding mode of a talin-vinculin-actin complex. The results here suggest that steric limitations between talin and actin should govern the exact order of binding events, or exact binding modes that would be adopted.

**Figure 10:**
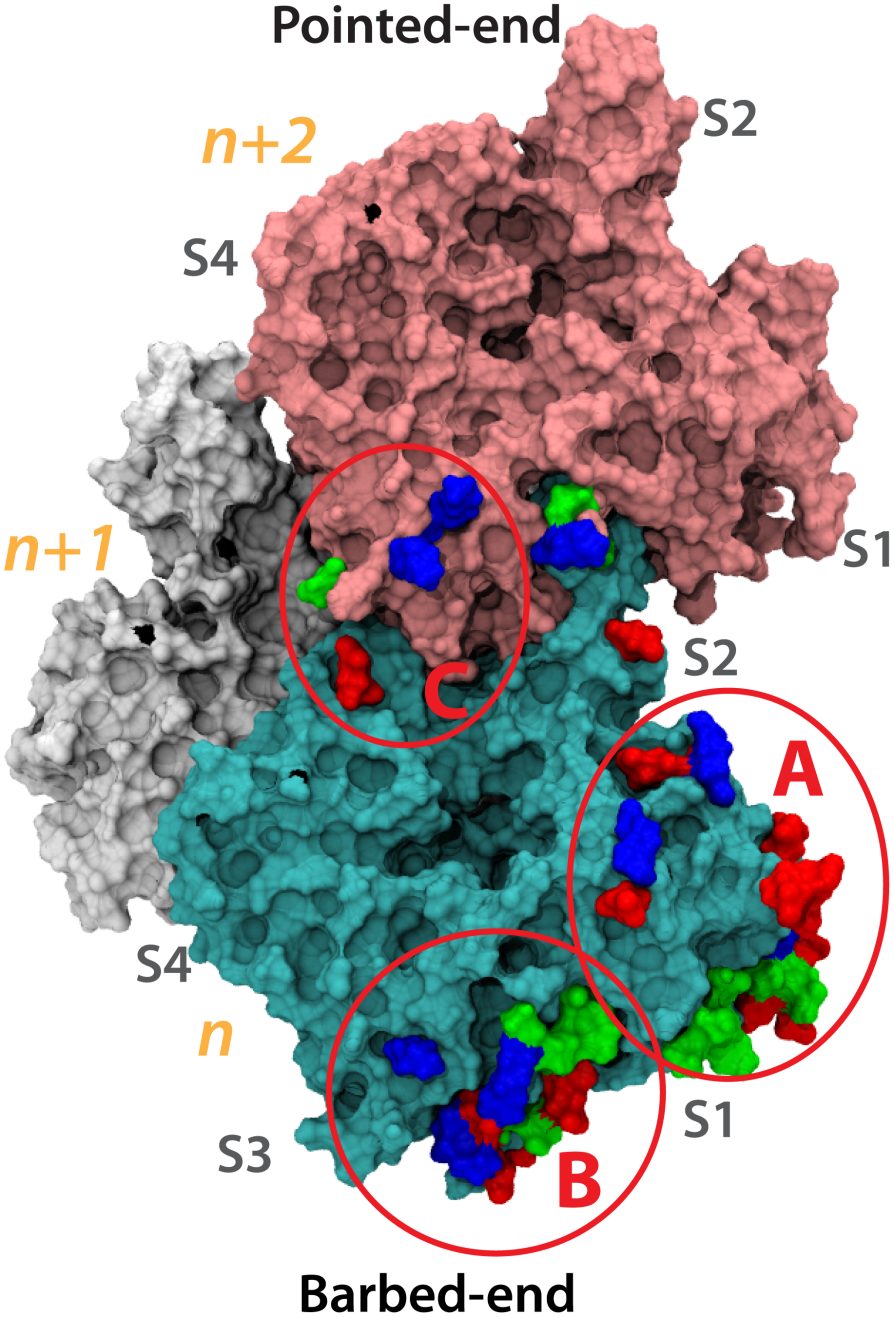
The vinculin binding regions on the surface of F-actin. The surface of F-actin is shown with all residues shown to interact vinculin colored by charge (basic residues in blue, acidic residues in red, and polar residues in green). The *n* subunit is shown in cyan, the *n-1* subunit in white, and the *n-2* subunit in pink. Three regions on the surface of F-actin are found to be involved in interaction with vinculin: (A) residues in S1 of subunit *n* are involved in both binding of vinculin along the filament and in capping of the filament by vinculin, (B) residues at S1 and S3 of the barbed-end are involved in capping of the filament by vinculin, and (C) residues at S3 of *n-2* and S4 of *n* are involved in binding to D1 of vinculin in its open conformation while binding F-actin along the filament.

Putting together the results from all of these simulations, we can predict three potential regions of an actin filament that would bind vinculin (Figure 10). Consistently, the acidic residues in S1 – vinculin binding site A – were shown to be critical for an interaction between Vt and F-actin. These residues stabilized both the binding of vinculin along the actin filament and they were involved in stabilizing Vt capping of the actin filament. The surface between S1 and S3 of the barbed end – vinculin binding site B – was also consistently shown to stabilize Vt. Hydrophobic residues in this region would form hydrophobic cores with non-polar residues from Vt. Both basic and acidic residues in this region would form salt-bridges with their counterparts on Vt. The interactions between Vt and S1 that were highlighted by our simulations had previously been suggested to be involved in binding of vinculin along the actin filament, and the interactions between S1 and S3 were previously shown to be significant for capping of the actin filament. However, the third region on the surface of the actin filament that is suggested here to be involved in vinculin binding is novel: the residues in S3 of subunit *n-2* – vinculin-binding site C. These residues can interact with charged residues from D1 of vinculin and in doing so contribute to further stabilizing the vinculin-actin linkage. With the presence of three binding regions we can predict that vinculin will differentially bind to each of the binding sites depending on the intensity of mechanical stress on the focal adhesion. It is possible that binding site A would interact with vinculin during vinculin activation, and would completely bind vinculin after activation. This interaction would require the least level of mechanical stress. Binding site C would bind vinculin after D1 is separated from Vt and can link to it. This would potentially require some level of mechanical stress. And binding of binding site B to vinculin would occur after transition of vinculin to the open II conformation. This would require the most level of mechanical stress. The exact binding interface between vinculin and F-actin, therefore, would be a function of the level of mechanical stress at the focal adhesion. Vinculin would be a variable switch at the focal adhesion, increasing its level of activation and F-actin binding depending on the level of mechanical stress at the focal adhesion.

**Figure 11:**
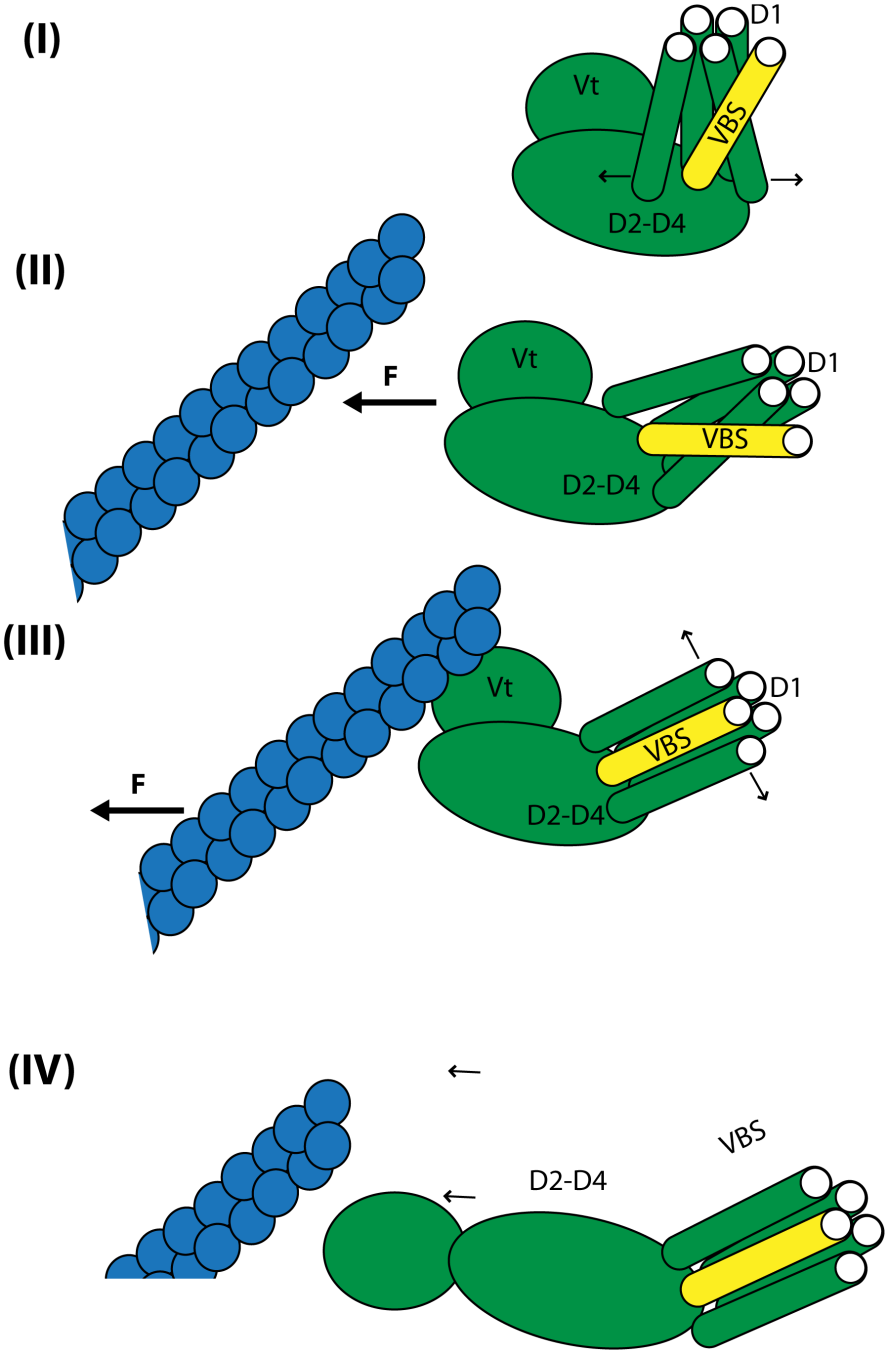
The open II conformation allows for dynamic linking to F-actin. The interaction of talin VBS with D1 of vinculin is regulated by the conformation of vinculin. (I) In its closed conformation vinculin can only link to talin VBS. (II) Transition of vinculin form a closed conformation to an open I conformation would result form interaction of Vt with F-actin combined with partial linking of D1 to talin. (III) Formation of the open I conformation allows for complete talin VBS insertion in to D1. (IV) Following formation of open I vinculin, further forces from F-actin can cause formation of the open II conformation. With the open II conformation vinculin can form a dynamic link to F-actin.

Previously, it was shown that formation of the vinculin open I conformation allows for the complete insertion of talin VBS in to D1 of vinculin [51]. We can now expand on those results and further state that after complete linking of vinculin to talin via D1, and linking along the actin filament via Vt in the open I conformation, any additional forces from further movement or stress of the actin filament could induce an open II conformation (Figure 11). The formation of an open II conformation would then allow vinculin to remain linked to F-actin even as it moves. In this way vinculin could act both as a molecular clutch and as a variable switch at the focal adhesion. The predictions from this study are especially relevant to understanding focal adhesion structures. Focal adhesions play a role in numerous cell types and are especially involved in the processes of cell migration. The predictions from this study contribute towards understanding molecular mechanisms of cell migration via the focal adhesions.

The question that remains unanswered after our simulations and analysis is whether the actin filaments can be capped at the focal adhesion. The simulations of vinculin in an open II conformation with the barbed-end suggested that F-actin can be capped, but what role would this play at the focal adhesion? The understanding that vinculin is a variable switch is a testable and valuable prediction from the molecular dynamics simulation, but the capping of actin filaments at focal adhesions is really an unanswered question that is posed by our molecular dynamics simulations. Further investigations – both computational and experimental – are sought to address this question.

## Methods

### Initial structure and configuration

PDB ID 1ST6 was used to build a structure of full-length vinculin [66]. The missing proline rich linker region (residues 843–877) was created via homology modeling using the SWISSMODEL toolkit [82], as previously described [42]. The proline rich linker region is suggested to be flexible and therefore its structure not resolved. Inclusion of a homology model for the linker region in this study is justified as the linker region is not suggested to play a key role in the binding events, and the simulations should not be affected by inclusion of the linker homology model. Vt was built as residues 895–1066 from the full-length vinculin model. The structure of open full-length vinculin was taken from previously published simulation [42]. PDB ID 3LUE [66] was used to build a structure of F-actin. The 3LUE structure has α-actinin [83] CH domains bound to F-actin. The CH-domains are removed and only 3 of the F-actin subunits are used to build the F-actin structure.

Complex of Vt bound to F-actin was build using Janssen et al [36] to orient Vt towards the two binding pockets along F-actin. Structure of full-length was build using the same Vt orientation but including the vinculin head domain residues. Vinculin was translated to be at least 15 Å away from F-actin. For simulation of Vt interaction with the barbed end, Vt was oriented either with the exposed or the occluded surface oriented towards Vt. Three arrangements of Vt with the barbed-end were simulated: Vt oriented towards S1 of the barbed-end of F-actin, Vt oriented towards S3 of the barbed-end of F-actin, Vt oriented towards both S1 and S3 of the barbed end. Vt was placed within 10 Å of the barbed-end in these structures. The initial orientation and setup of each simulation was random with respect to the exact pose of vinculin relative to actin; the distance from actin was imposed to be 10 Å and the face of vinculin (exposed or occluded surface) closest to actin was controlled, but the exact orientation pose of vinculin was chosen at random. It is possible the orientation pose of vinculin directly impacted the final binding mode, but given that the final binding orientation and pose of vinculin bound to actin was government by the mechanics of interaction, the final binding mode can be seen as more representative. For simulation with vinculin in the closed or the open II conformation, additional vinculin head residues are included with maintaining the Vt orientation. Each system was solvated with 12 Å of padding at each end of the simulation box.

### Molecular dynamic simulation

Simulations were carried out using the NAMD Scalable Molecular Dynamics program [84], using an explicit solvent representation. Periodic boundary conditions were used along with a Langevin piston Nose-Hoover [85] mechanism for pressure control at 1 Atm. Constant temperature of 310K was maintained using a Langevin damping coefficient of 5/ps. Rigid bonds were enforced between hydrogen atoms and their bound larger atoms [86]. The CHARMm 27 force fields were used [87], [88]. Simulation timesteps of 2 fs were used for all molecular dynamics.

Each configuration was first minimized for 1000 steps using the conjugate gradient and line search algorithm implemented in NAMD [84]. Following minimization each configuration is simulated for at least 15 ns or until equilibration. All simulation results were visualized and analyzed using VMD [89]. For simulations of binding along F-actin the Vt, closed vinculin, and open I vinculin were initially nudged towards F-actin for less than 1 ns prior to simulation for 15 ns using a constant velocity pull on the center of mass of vinculin in a direction towards the center of mass of actin. Use of the nudge reduces the entropic barrier to binding. For simulation of Vt capping actin filaments no initial nudge is used and instead Vt is placed 5 Å closer to the barbed-end. Vt is smaller than full-length vinculin and can have faster translation, thus binding occurred even without an initial nudging force. The simulations are performed one time per setup. Additional simulations would allow for a statistical estimation of reproducibility, however, given computational limitations to running multiple 15 ns simulations, the present study is limited to a single simulation per setup.

### Umbrella sampling

Umbrella sampling of D4 separation from Vt was carried out using GROMACS [90]. The umbrella sampling approach allows estimation of a free energy path along a reaction coordinate by estimation of the free energy difference between subsections of the path. The reaction coordinate was defined as the distance between the center of mass of D4 and the center of mass of Vt. Residues in D1 16, 51, 81, and 115 were constrained with 1000 KJ/mol*nm^2^ to maintain an open I conformation throughout the simulation. Residues 926, 958, 988, and 1031 of Vt were defined as the pull group and constrained along the reaction coordinate away from residues 730, 760, 794, and 824 of D4. An umbrella potential of 1000 KJ/mol*nm^2^ was used with a reference step of 0.2 Å in order to maximize umbrella overlap, allowing for an accurate estimation of the free energy path. The final potential of mean force was calculated using Grossfield's WHAM code [91].

## Supporting Information

Figure S1 **Distance between interacting residues on Vt and F-actin.** Simulation of Vt interaction with F-actin showed linking of Vt to F-actin. The distance between 9 residues on Vt and their respective interacting residues on F-actin is tracked throughout the 15 ns simulation. 8 interactions show clear association by the end of the simulations. Two interactions, E986 with R28 and T1004 with K328, show intermittent association and then dissociation.
(PDF)

Figure S2 **Potential energy changes resulting from interaction of Vt with F-actin.** Simulation of Vt interacting with F-actin showed the final bound complex consists of two binding regions: (1) the interaction of Vt with actin subunit *n-2*, and (2) the interaction of Vt with actin subunit *n*. The potential energy between the binding residues on Vt and on F-actin are calculated throughout the 15 ns simulation. The potential energy between Vt and *n-2* is shown in green and the potential energy between Vt and *n* is shown in red. The interaction of Vt with *n* results in a 150 Kcal/mol reduction in the potential energy of the interacting complex, whereas the interaction of Vt with *n-2* produces negligible change in the potential energy. A loss in potential energy results from interaction between the basic residues on Vt and the acidic residues on F-actin and could represent formation of a stable interaction.
(PDF)

Figure S3 **Distance between interacting residues on closed vinculin and F-actin.** Simulation of full-length vinculin in the closed conformation with F-actin showed no linkage between vinculin and actin. The distance between 8 residues on vinculin and their respective residues near to them on actin are tracked throughout the 15 ns simulation. All residues showed consistent separation through the simulation. No residues formed stable linkages.
(PDF)

Figure S4 **Distance between interacting residues on open vinculin and F-actin.** Simulation of open vinculin with F-actin showed linkage between vinculin and F-actin. The distance between 12 residues on vinculin and their respective interacting residues on F-actin are tracked and plotted. Within 3 ns all linkages form a minimum and continue to equilibrate around that minimum over the next 12 ns of simulation. No linkages showed dissociation during the simulation.
(PDF)

Figure S5 **Potential energy changes resulting from interaction of full-length vinculin with F-actin.** The potential energy between binding residues on vinculin and on F-actin is calculated for both simulation of binding along the filament using the closed conformation of vinculin and using the suggested open conformation of vinculin. The interaction between vinculin in the closed conformation and F-actin resulted in steric clashes and no binding. The potential energy between clashing residues is plotted in red. Since no binding occurred and the closed vinculin moved away from F-actin no potential energy is reported between the two. Vinculin in a open conformation forms interactions between Vt and the *n* subunit and between D1 and the *n-2* subunit. The potential energy between interacting residues in both sets of interactions is plotted: the energy between D1 and *n-2* is plotted in blue; the energy between Vt and *n* is plotted in orange. Interaction of Vt with *n* resulted in a reduction of potential energy of up to 200 Kcal/mol. In contrast the interaction between D1 and *n-2* resulted in no more than a 50 Kcal/mol reduction in potential energy. The link between Vt and *n* is the most energetically favorable of the interactions.
(PDF)

Figure S6 **Interaction along the actin filament by three vinculin structures.** Three simulations were produced of vinculin interacting with actin along its filament: (A) using the structure of Vt only, (B) using the structure of vinculin in its closed conformation, and (C) using the structure of vinculin in a suggested open conformation. The final arrangement of the molecules is shown from each of the three simulations after 15 ns of simulation with the same viewpoint. Only interaction of Vt (A) and interaction vinculin in its open conformation (C) resulted in a stable linkage along the actin filament. Interaction of vinculin in its closed conformation (B) resulted in steric repulsion.
(PDF)

Figure S7 **Distance between interacting residues on Vt (exposed) and S1 of the actin barbed-end.** Simulation of Vt with the barbed-end of F-actin while approaching S1 with its exposed surface showed linkage between Vt and F-actin. The distance between 10 Vt residues and their respective interacting residues on S1 are tracked and plotted. All 10 residues show association within 11 ns of simulation and remain associated throughout the last 4 ns of simulation.
(PDF)

Figure S8 **Distance between interacting residues on Vt (occluded) and S1 of the actin barbed-end.** Simulation of Vt with the barbed-end of F-actin while approaching S1 with its occluded surface showed linkage between Vt and F-actin. The distance between 12 Vt residues and their respective interacting residues on S1 are tracked and plotted. All 12 residues show association within 10 ns of simulation and remain associated throughout the last 5 ns of simulation.
(PDF)

Figure S9 **Comparison of potential energy changes from capping S1 by exposed and occluded Vt.** The potential energy between binding residues on S1 and on Vt is calculated for both simulations with the exposed surface of Vt oriented towards S1 and with the occluded surface of Vt oriented towards S1. In both simulations the potential energy is reduced by as much as 350 Kcal/mol as the simulation progresses and the two molecules are linked together. Changes in the potential energy throughout the 15 ns simulation with the exposed surface of vinculin is plotted in blue, and the potential energy throughout the simulation with the occluded surface of vinculin is plotted in red. Binding at both surfaces to S1 are energetically highly favorable.
(PDF)

Figure S10 **Distance between interacting residues on Vt (exposed) and S3 of the actin barbed-end.** Simulation of Vt with the barbed-end of F-actin while approaching S3 with its exposed surface showed little linkage between Vt and F-actin. The distance between 3 Vt residues and their respective interacting residues in S3 are tracked and plotted. The Cα of all 3 residues are within 12 Å after 9 ns of simulation and are close enough to be associating. The linkages are not as strong as between Vt and S1.
(PDF)

Figure S11 **Distance between interacting residues on Vt (occluded) and S3 of the actin barbed-end.** Simulation of Vt with the barbed-end of F-actin while approaching S3 with its occluded surface showed little linkage between Vt and F-actin. The distance between 3 Vt residues and their respective interacting residues in S3 are tracked and plotted. The three residues remain within 10 Å of each but do not associate any closer.
(PDF)

Figure S12 **Comparison of potential energy changes from S1 capping by exposed and occluded Vt.** The potential energy between binding residues in Vt and S1 of the capping-end of F-actin is calculated throughout the 15 ns simulations of both interaction with the exposed surface of Vt and interaction with the occluded surface of Vt. The potential energy of the exposed surface's interaction is plotted in green and the potential energy of the occluded surface's interaction is plotted in blue. Interaction with the exposed surface never reduces the potential energy between the binding residues more than 150 Kcal/mol whereas interaction with the occluded surface of Vt reduced the potential energy between the binding residues by as much as 250 Kcal/mol. Both interactions are favorable, but the interaction with the occluded surface is more stabilizing as it reduces the potential energy further.
(PDF)

Figure S13 **Distance between interacting residues on Vt (exposed) and both S1 and S3 of the actin barbed-end.** Simulation of Vt with the barbed-end of F-actin while approaching both S1 and S3 with its exposed surface showed some linkage. The distance between 3 Vt residues and their respective interacting residues on S1 and S3 are tracked and plotted. Two interaction are stable and link Vt to both S1 and S3, one interaction, E960 and Q360, was intermittent and dissociated after 10 ns of simulation.
(PDF)

Figure S14 **Distance between interacting residues on Vt (occluded) and both S1 and S3 of the actin barbed-end.** Simulation of Vt with the barbed-end of F-actin while approaching both S1 and S3 with its exposed surface showed strong linkage. The distance between 12 Vt residues and their respective interacting residues on S1 and S3 are tracked and plotted. All 12 interactions showed stable linkage within 10 ns of simulation and remained linked for the remaining 5 ns of the simulation. This interaction was the strongest of the simulations of Vt interacting with the barbed-end of F-actin.
(PDF)

Figure S15 **Comparison of potential energy changes from capping S1 and S3 by exposed and occluded Vt.** The potential energy between binding residues from Vt and S1 and S3 of the barbed-end of F-actin is calculated throughout the 15 ns simulation for both the interaction of the exposed surface of Vt with S1 and S3 and the interaction of the occluded surface of Vt with S1 and S3. The interaction of Vt using its exposed surface with F-actin reduces the potential energy of the complex by 100 Kcal/mol. The interaction of Vt using it occluded surface with F-actin reduces the potential energy of the complex by over 550 Kcal/mol. The interaction between the occluded surface of Vt and the S1 and S3 subdomains is highly energetically favorable and likely represents the true binding interface between Vt and the barbed-end of F-actin.
(PDF)

Figure S16 **F-actin capping by Vt was investigated with six possible arrangements.** A total of six possible arrangements of Vt and F-actin were simulated to investigate the interaction between vinculin and F-actin. Two surfaces of Vt were used, the surface exposed to solvent and F-actin (A), (B), and (C), and the surface normally occluded from F-actin by the vinculin head (D), (E), and (F). Both surfaces were initially arranged such that they were oriented towards S1 (C) and (F), towards S3 (A) and (D), or towards both S1 and S3 (B) and (E); exposed and occluded surfaces respectively. Interaction of Vt with either S1 only or with S1 and S3 was found to be more stable. Interaction with the occluded surface of Vt was found to be more likely than interaction with the exposed surface of Vt. Vt is shown in orange. Subunit *n* is shown in blue, and subunit *n-2* is shown in red.
(PDF)

Figure S17 **The interactions between D4 and Vt break to allow for the open II conformation.** To produce the open II conformation, Vt is pulled away from D4. There are 5 salt-bridges between D4 and Vt: R1057 with D847, R1060 with D856, R978 with K1047, K975 with E775, and R976 with E770. The presence of these salt-bridges stabilized Vt near to the vinculin head and likely prevents interaction of Vt with the barbed-end of F-actin. After these interactions are broken and Vt separated from D4 and the rest of the vinculin head domain, then Vt is likely to be able to bind the barbed-end of the actin filaments.
(PDF)

Figure S18 **Potential energy between interacting residues in Vt and D4 during open II formation.** The potential energy between residues interacting on Vt and D4 is calculated throughout the simulation of open II formation. The first 5 ns of the simulation are plotted here. The interactions included in the calculation are: R1057 with D841, R1060 with D856, K1047 and R976 with E770, R978 with E775, and K975 with E770. Initially there is over 500 Kcal/mol of potential energy stored in the interaction between Vt and D4. As Vt separates during formation of the open II conformation the stored energy is relieved. Within 2 ns of simulation the interactions are broken.
(PDF)

Figure S19 **Distance between interacting residues on closed vinculin and F-actin.** Simulation of full-length vinculin in its closed conformation with the barbed-end of F-actin while approaching both S1 and S3 showed little linkage. The distance between 3 residues on vinculin and their respective interacting residues on S1 and S3 are tracked and plotted. All 3 interactions did interact favorable. By the end of the 15 ns of simulation they are closer than 10 Å from each other.
(PDF)

Figure S20 **Comparison of the potential energy change after capping by two vinculin conformations.** The potential energy between interacting residues on vinculin and the barbed-end of F-actin are calculated throughout 15 ns of simulation for both simulation with the closed conformation and simulation with a conformational change releasing Vt from the vinculin head. The potential energy between interacting residues is never reduced more than 50 Kcal/mol in interaction with the closed conformation of vinculin (blue plot). In contrast, interaction of vinculin after a second conformational change with S1 and S3 reduces the potential energy of the interacting residues by as much as 300 Kcal/mol. This interaction is highly energetically favorable and likely represents the true interface formed by capping of F-actin by full-length vinculin.
(PDF)

Figure S21 **Distance between interacting residues on open II vinculin and F-actin.** Simulation of full-length vinculin in its open II conformation with the barbed-end of F-actin while approaching both S1 and S3 showed strong linkage. The distance between 8 residues on Vt and their respective interacting residues on S1 and S3 are tracked and plotted. All 8 interactions show a linkage and a decrease in distances. The decrease in distances are not as large as with simulation of Vt alone.
(PDF)
