# Supplementary figures and images for: The Interaction of Vinculin with Actin
Source: PLoS Comput Biol. 2013 Apr 25;9(4):e1002995. doi: 10.1371/journal.pcbi.1002995 (PMC3635976; doi:10.1371/journal.pcbi.1002995)

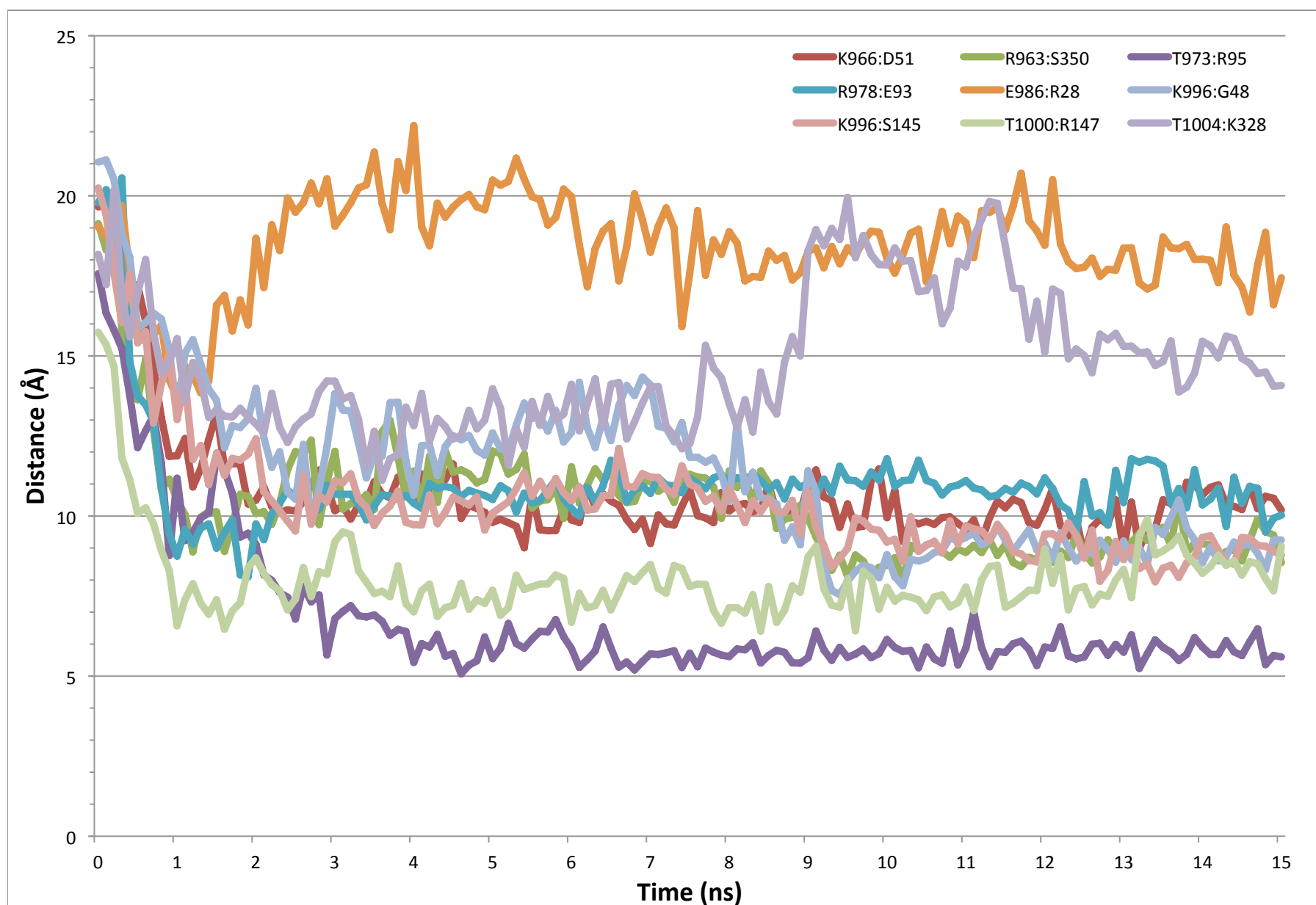

Supplement: Figure S1 — Distance between interacting residues on Vt and F-actin. Simulation of Vt interaction with F-actin showed linking of Vt to F-actin. The distance between 9 residues on Vt and their respective interacting residues on F-actin is tracked throughout the 15 ns simulation. 8 interactions show clear association by the end of the simulations. Two interactions, E986 with R28 and T1004 with K328, show intermittent association and then dissociation. (PDF) [file pcbi.1002995.s001.pdf]

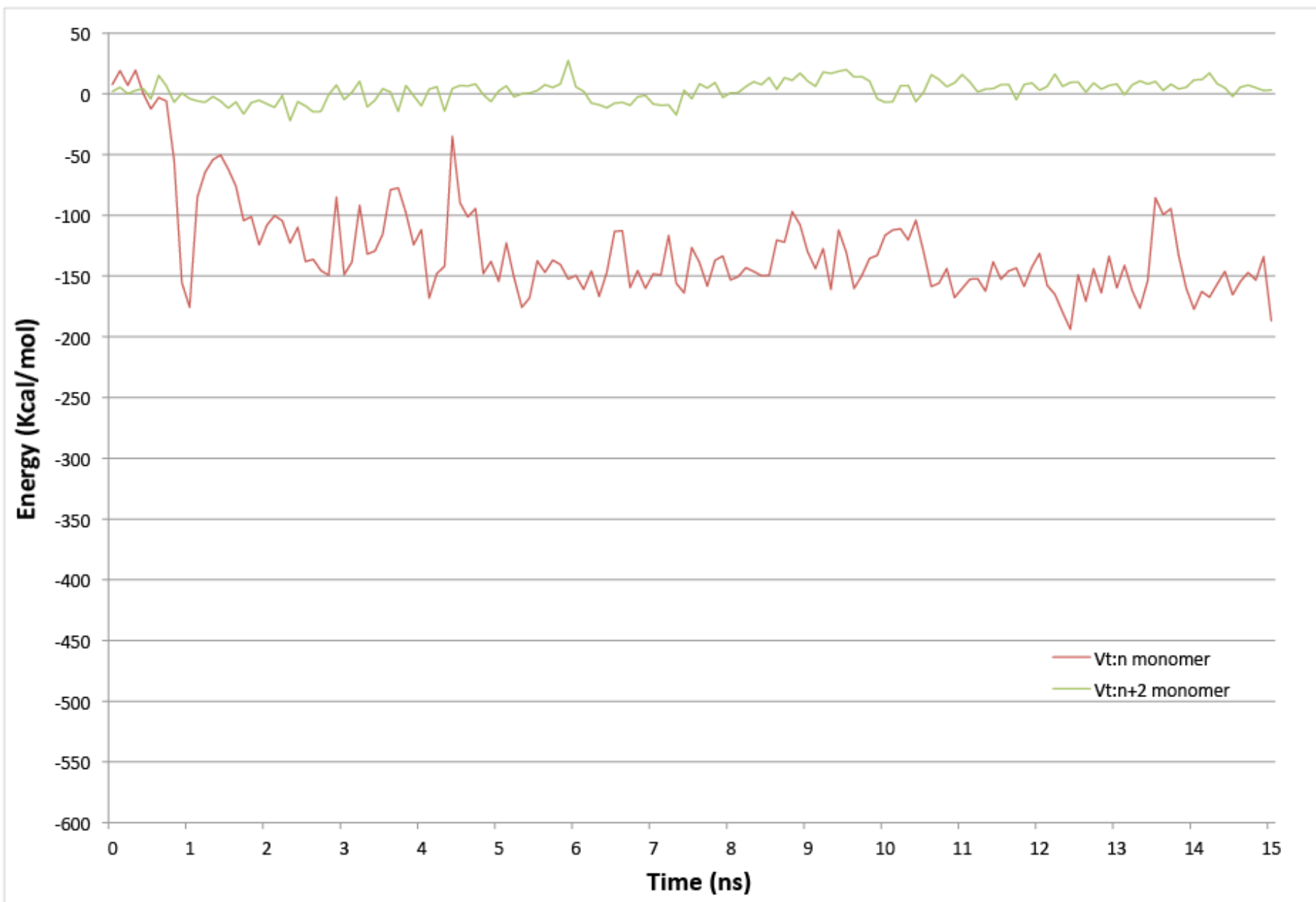

Supplement: Figure S2 — Potential energy changes resulting from interaction of Vt with F-actin. Simulation of Vt interacting with F-actin showed the final bound complex consists of two binding regions: (1) the interaction of Vt with actin subunit n-2, and (2) the interaction of Vt with actin subunit n. The potential energy between the binding residues on Vt and on F-actin are calculated throughout the 15 ns simulation. The potential energy between Vt and n-2 is shown in green and the potential energy between Vt and n is shown in red. The interaction of Vt with n results in a 150 Kcal/mol reduction in the potential energy of the interacting complex, whereas the interaction of Vt with n-2 produces negligible change in the potential energy. A loss in potential energy results from interaction between the basic residues on Vt and the acidic residues on F-actin and could represent formation of a stable interaction. (PDF) [file pcbi.1002995.s002.pdf]

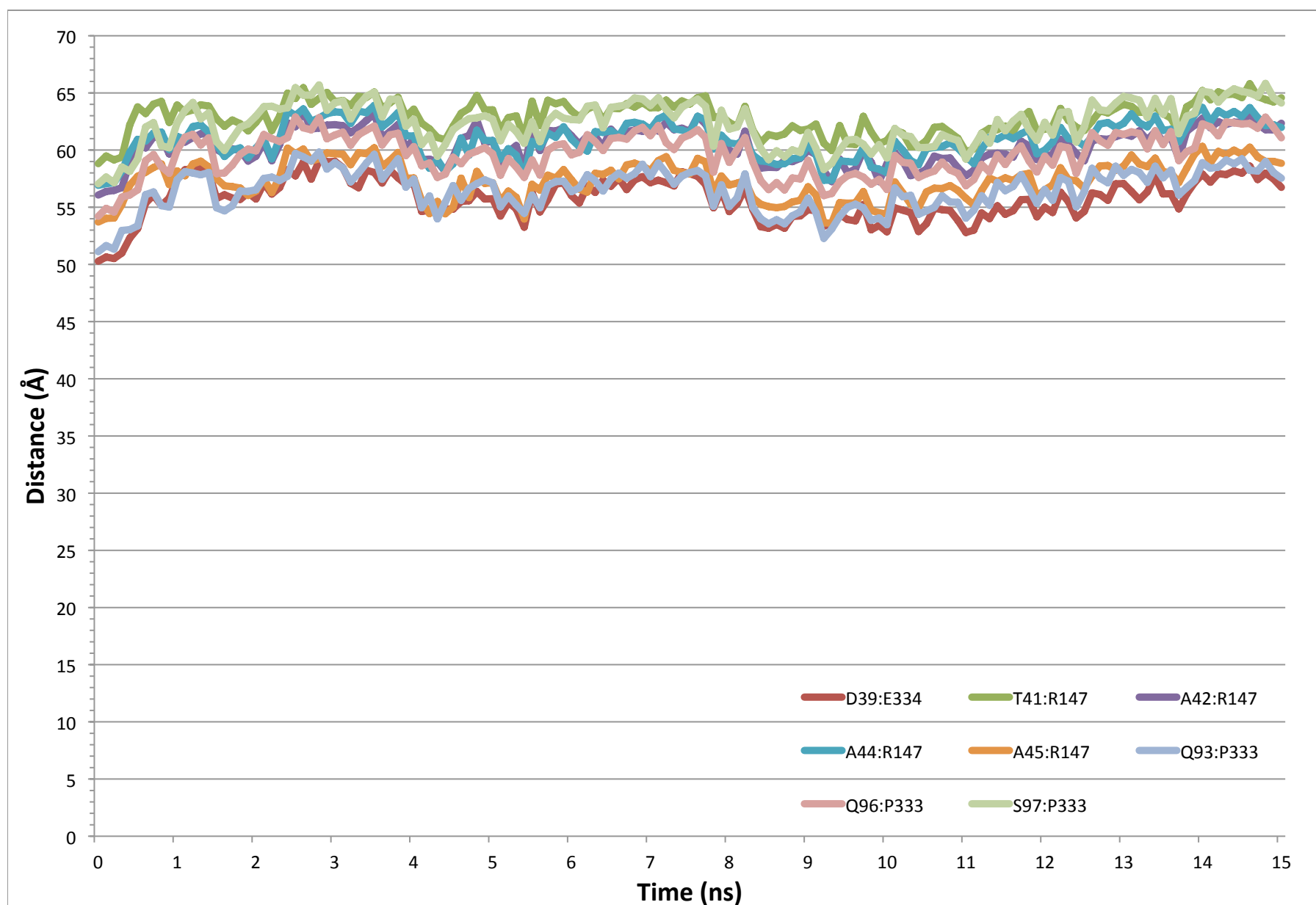

Supplement: Figure S3 — Distance between interacting residues on closed vinculin and F-actin. Simulation of full-length vinculin in the closed conformation with F-actin showed no linkage between vinculin and actin. The distance between 8 residues on vinculin and their respective residues near to them on actin are tracked throughout the 15 ns simulation. All residues showed consistent separation through the simulation. No residues formed stable linkages. (PDF) [file pcbi.1002995.s003.pdf]

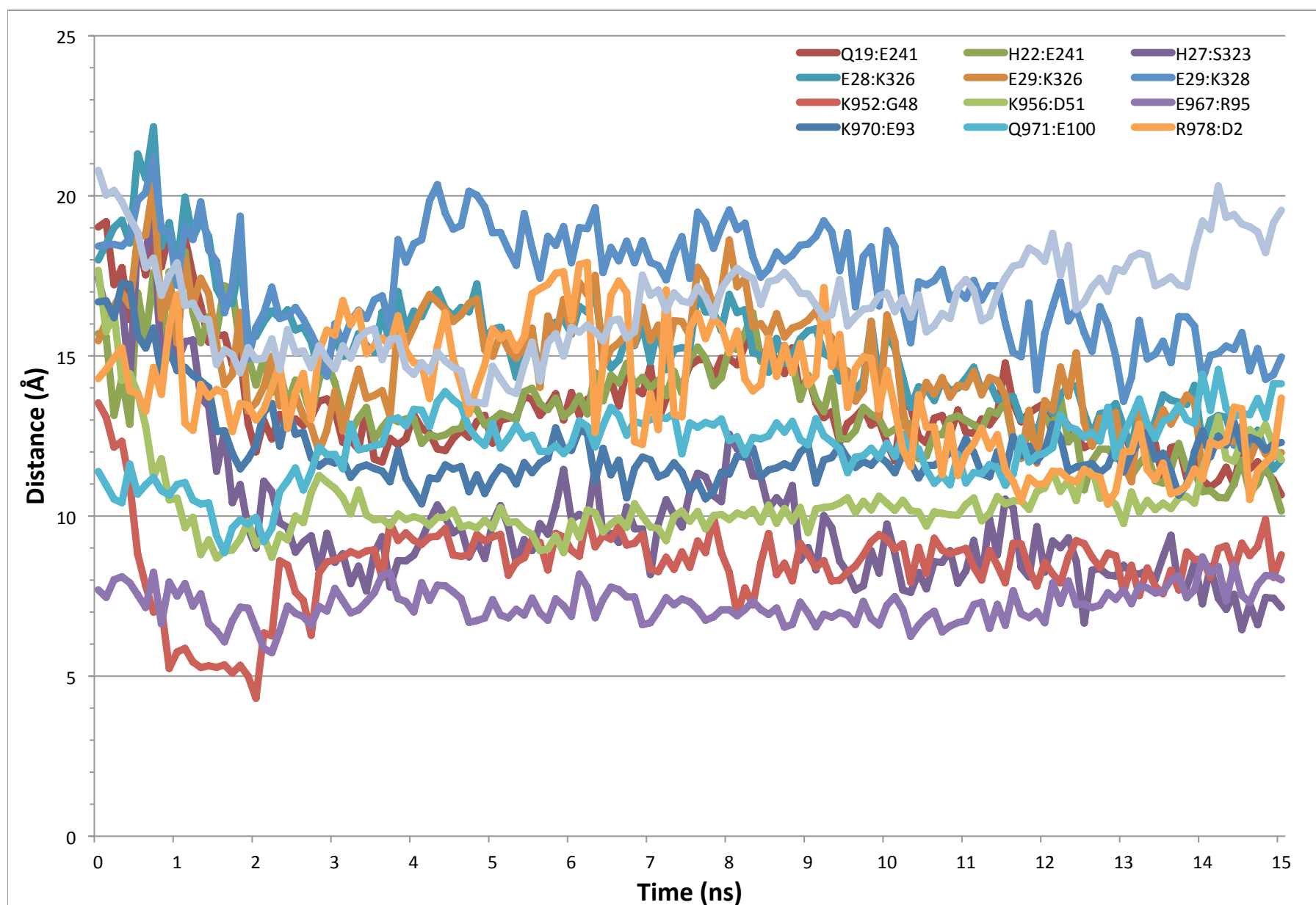

Supplement: Figure S4 — Distance between interacting residues on open vinculin and F-actin. Simulation of open vinculin with F-actin showed linkage between vinculin and F-actin. The distance between 12 residues on vinculin and their respective interacting residues on F-actin are tracked and plotted. Within 3 ns all linkages form a minimum and continue to equilibrate around that minimum over the next 12 ns of simulation. No linkages showed dissociation during the simulation. (PDF) [file pcbi.1002995.s004.pdf]

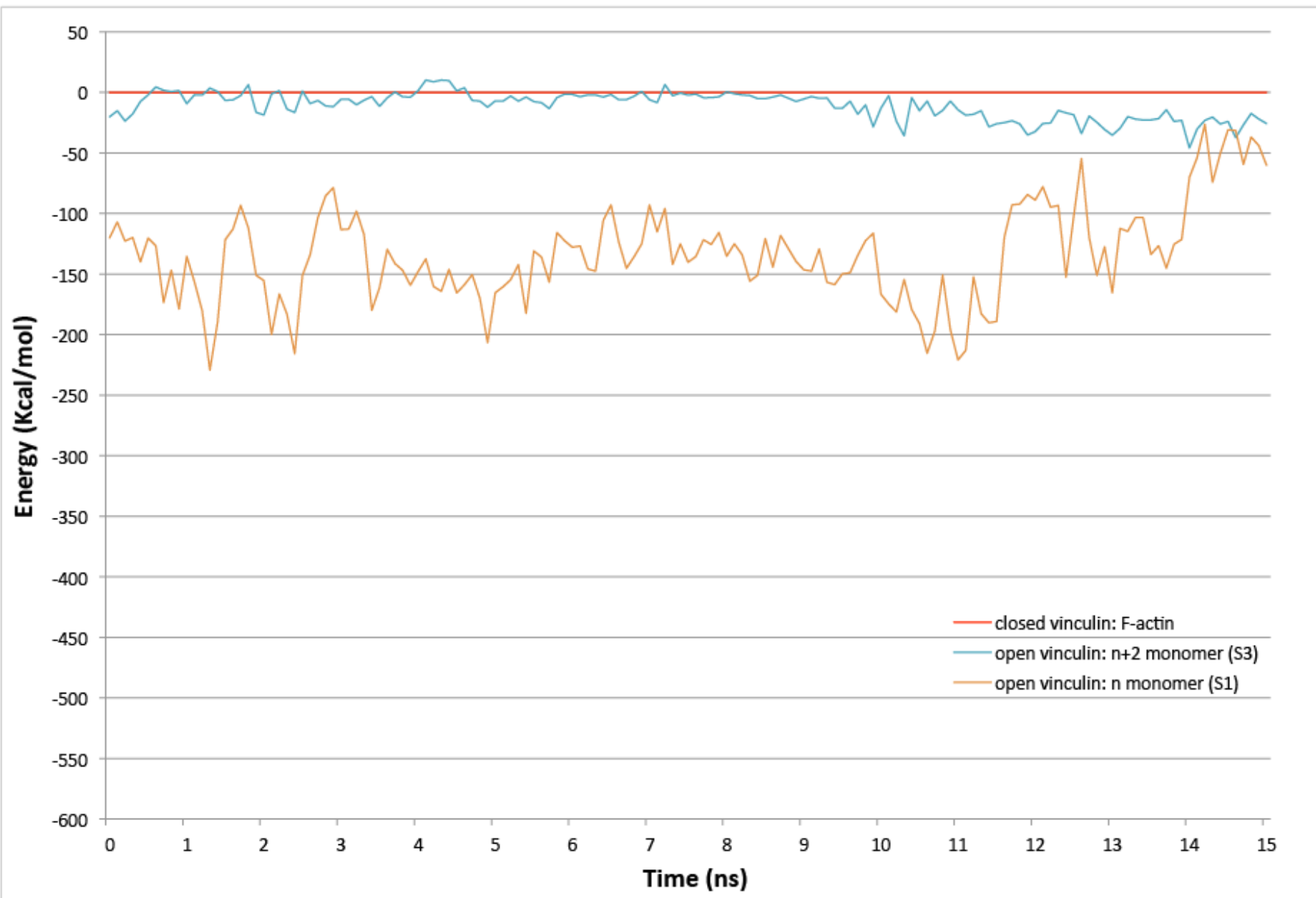

Supplement: Figure S5 — Potential energy changes resulting from interaction of full-length vinculin with F-actin. The potential energy between binding residues on vinculin and on F-actin is calculated for both simulation of binding along the filament using the closed conformation of vinculin and using the suggested open conformation of vinculin. The interaction between vinculin in the closed conformation and F-actin resulted in steric clashes and no binding. The potential energy between clashing residues is plotted in red. Since no binding occurred and the closed vinculin moved away from F-actin no potential energy is reported between the two. Vinculin in a open conformation forms interactions between Vt and the n subunit and between D1 and the n-2 subunit. The potential energy between interacting residues in both sets of interactions is plotted: the energy between D1 and n-2 is plotted in blue; the energy between Vt and n is plotted in orange. Interaction of Vt with n resulted in a reduction of potential energy of up to 200 Kcal/mol. In contrast the interaction between D1 and n-2 resulted in no more than a 50 Kcal/mol reduction in potential energy. The link between Vt and n is the most energetically favorable of the interactions. (PDF) [file pcbi.1002995.s005.pdf]

**A**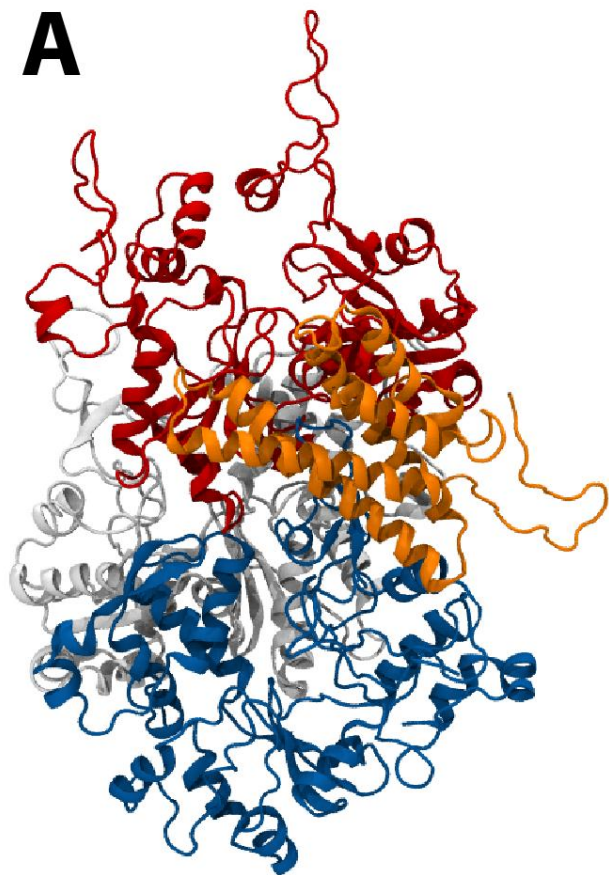**B**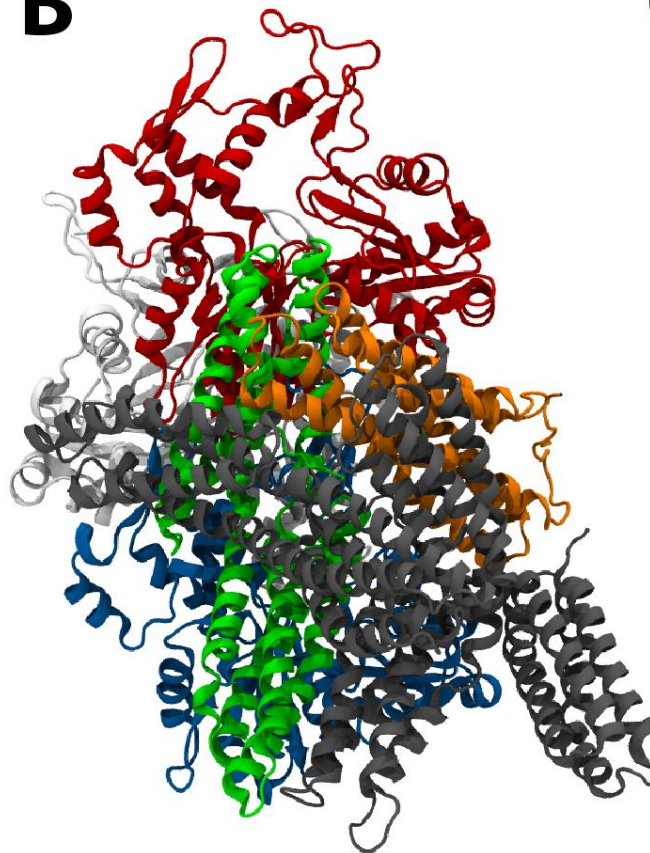**C**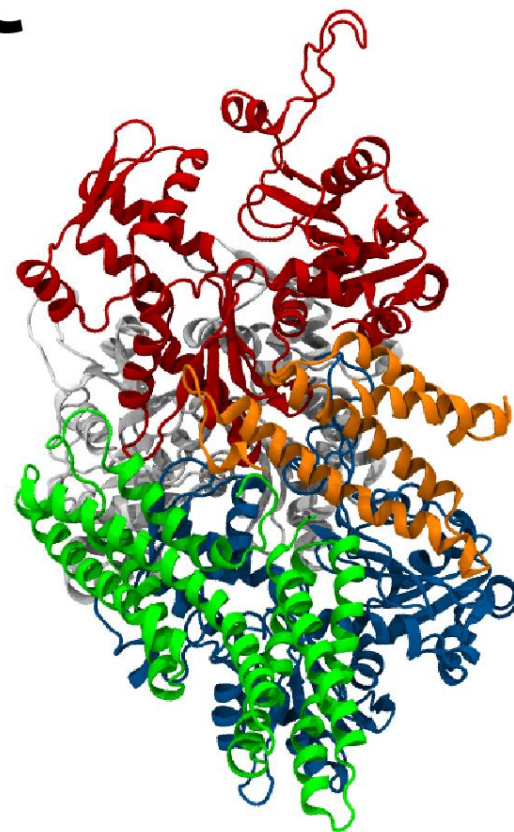

Supplement: Figure S6 — Interaction along the actin filament by three vinculin structures. Three simulations were produced of vinculin interacting with actin along its filament: (A) using the structure of Vt only, (B) using the structure of vinculin in its closed conformation, and (C) using the structure of vinculin in a suggested open conformation. The final arrangement of the molecules is shown from each of the three simulations after 15 ns of simulation with the same viewpoint. Only interaction of Vt (A) and interaction vinculin in its open conformation (C) resulted in a stable linkage along the actin filament. Interaction of vinculin in its closed conformation (B) resulted in steric repulsion. (PDF) [file pcbi.1002995.s006.pdf]

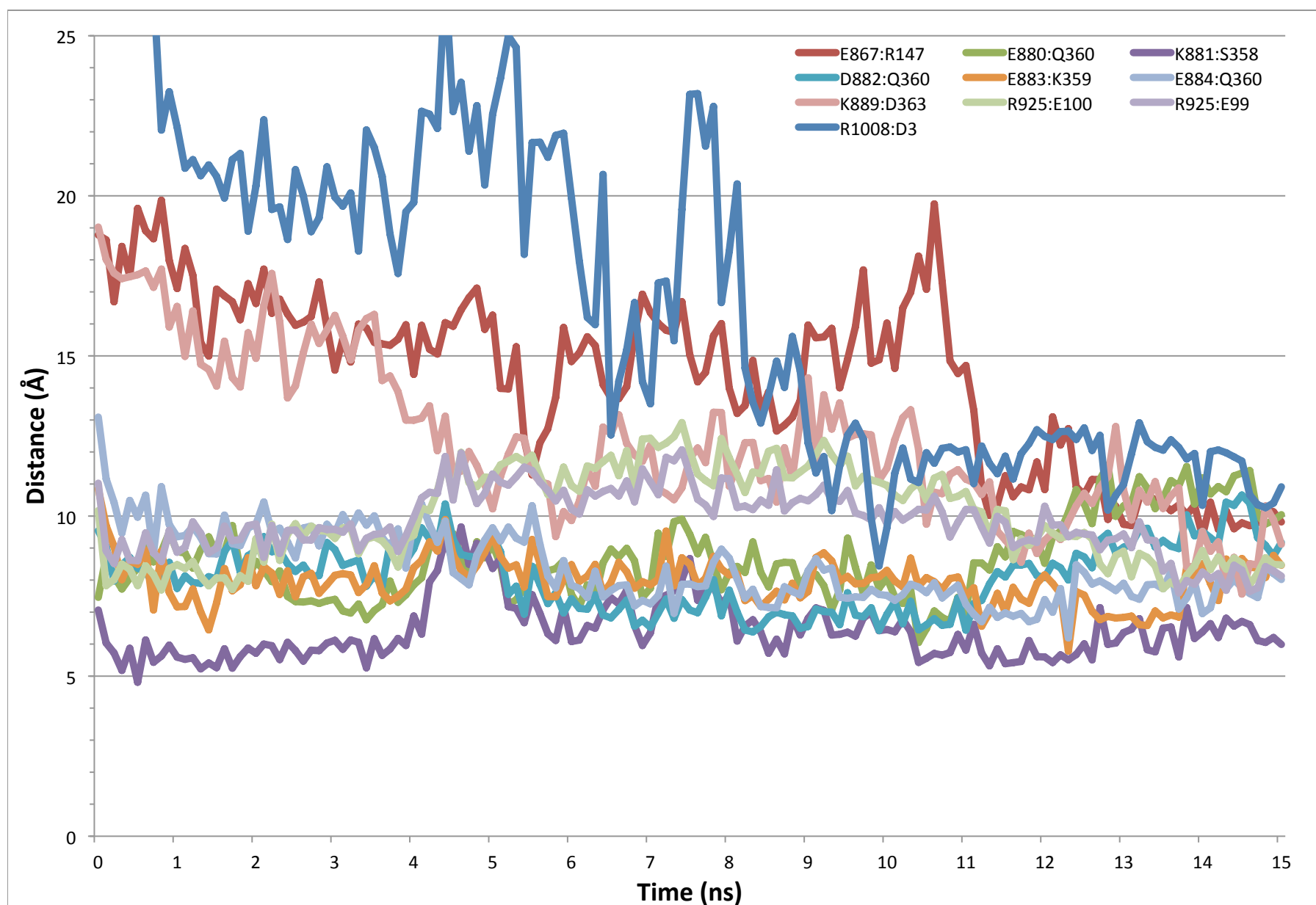

Supplement: Figure S7 — Distance between interacting residues on Vt (exposed) and S1 of the actin barbed-end. Simulation of Vt with the barbed-end of F-actin while approaching S1 with its exposed surface showed linkage between Vt and F-actin. The distance between 10 Vt residues and their respective interacting residues on S1 are tracked and plotted. All 10 residues show association within 11 ns of simulation and remain associated throughout the last 4 ns of simulation. (PDF) [file pcbi.1002995.s007.pdf]

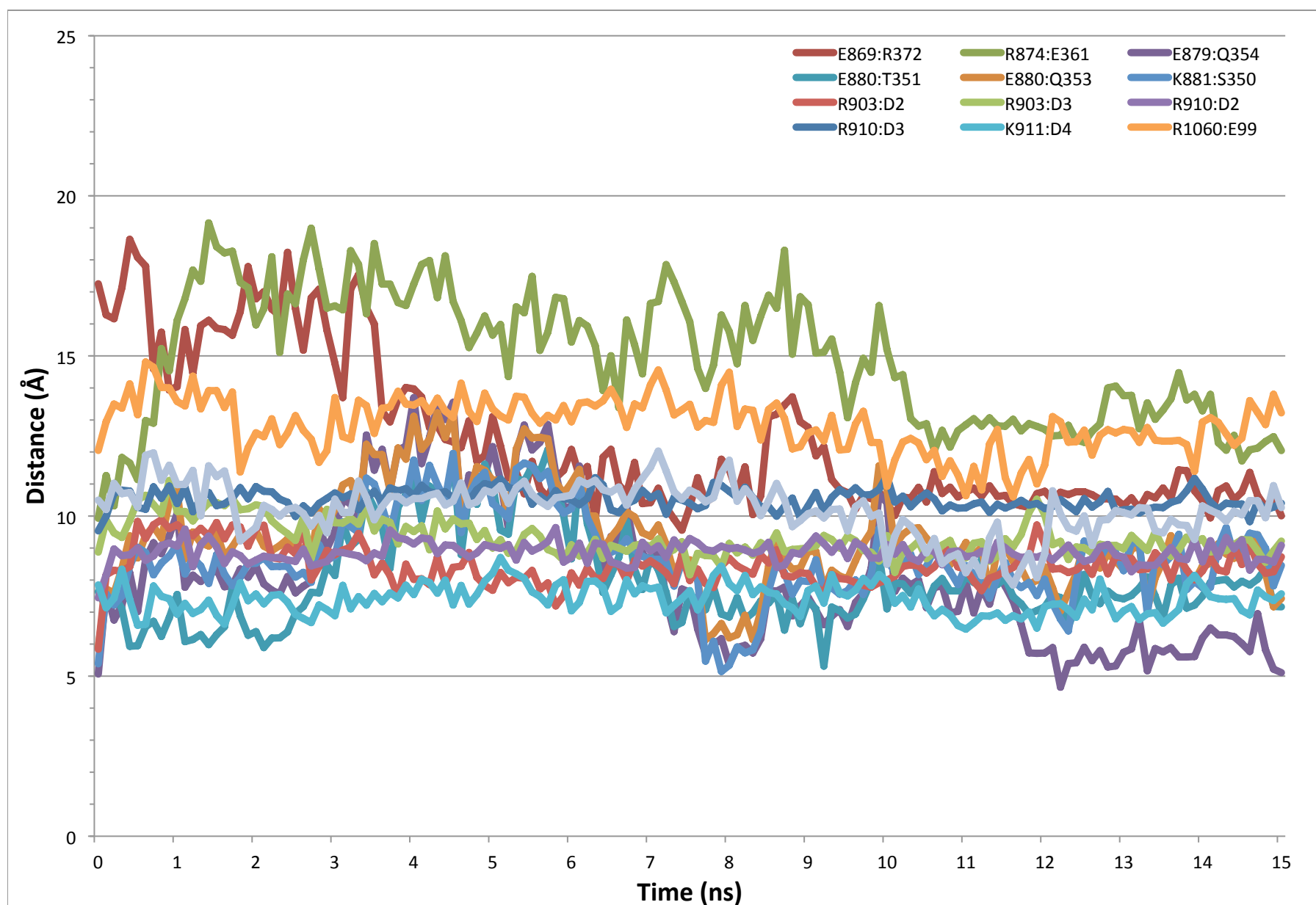

Supplement: Figure S8 — Distance between interacting residues on Vt (occluded) and S1 of the actin barbed-end. Simulation of Vt with the barbed-end of F-actin while approaching S1 with its occluded surface showed linkage between Vt and F-actin. The distance between 12 Vt residues and their respective interacting residues on S1 are tracked and plotted. All 12 residues show association within 10 ns of simulation and remain associated throughout the last 5 ns of simulation. (PDF) [file pcbi.1002995.s008.pdf]

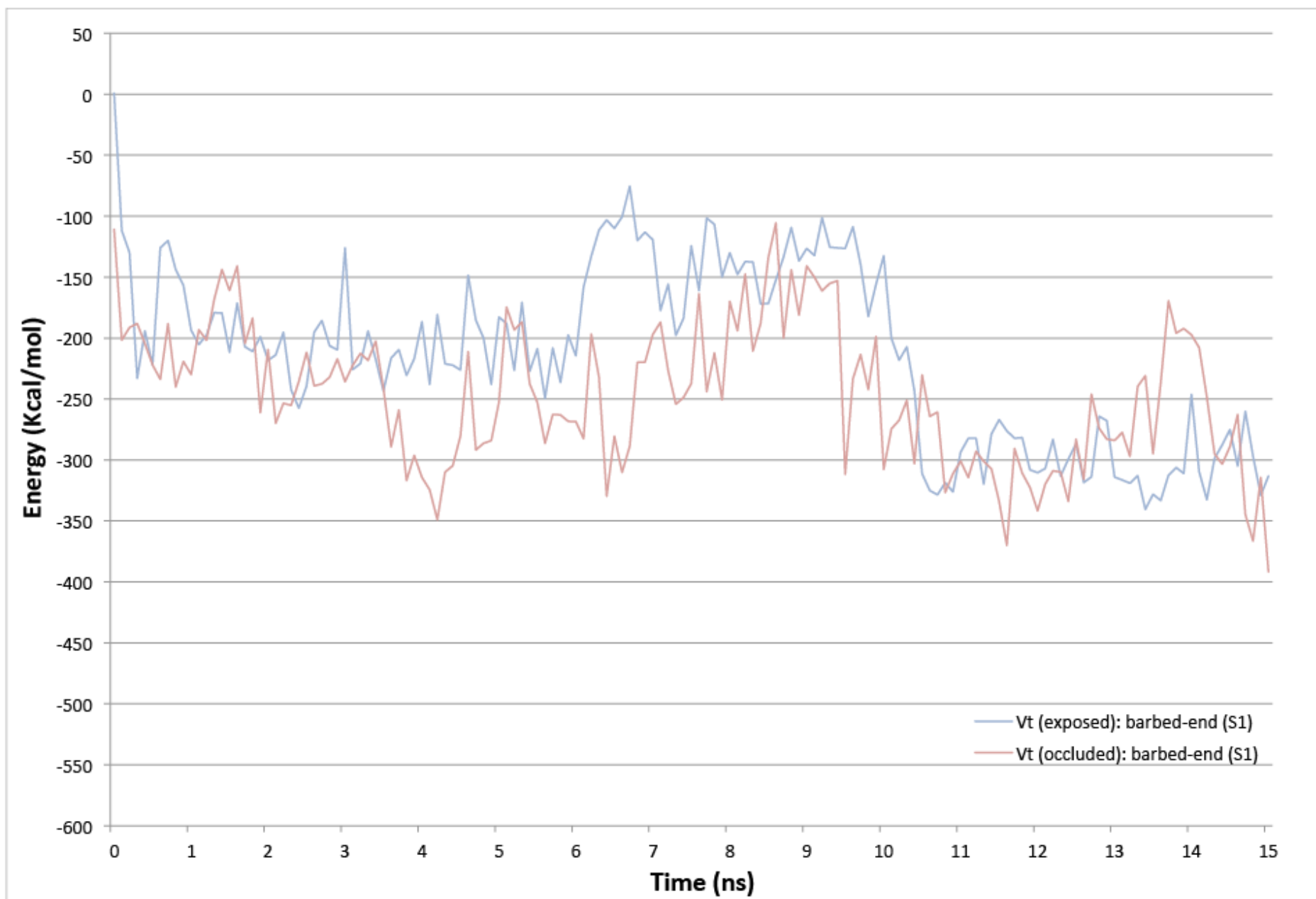

Supplement: Figure S9 — Comparison of potential energy changes from capping S1 by exposed and occluded Vt. The potential energy between binding residues on S1 and on Vt is calculated for both simulations with the exposed surface of Vt oriented towards S1 and with the occluded surface of Vt oriented towards S1. In both simulations the potential energy is reduced by as much as 350 Kcal/mol as the simulation progresses and the two molecules are linked together. Changes in the potential energy throughout the 15 ns simulation with the exposed surface of vinculin is plotted in blue, and the potential energy throughout the simulation with the occluded surface of vinculin is plotted in red. Binding at both surfaces to S1 are energetically highly favorable. (PDF) [file pcbi.1002995.s009.pdf]

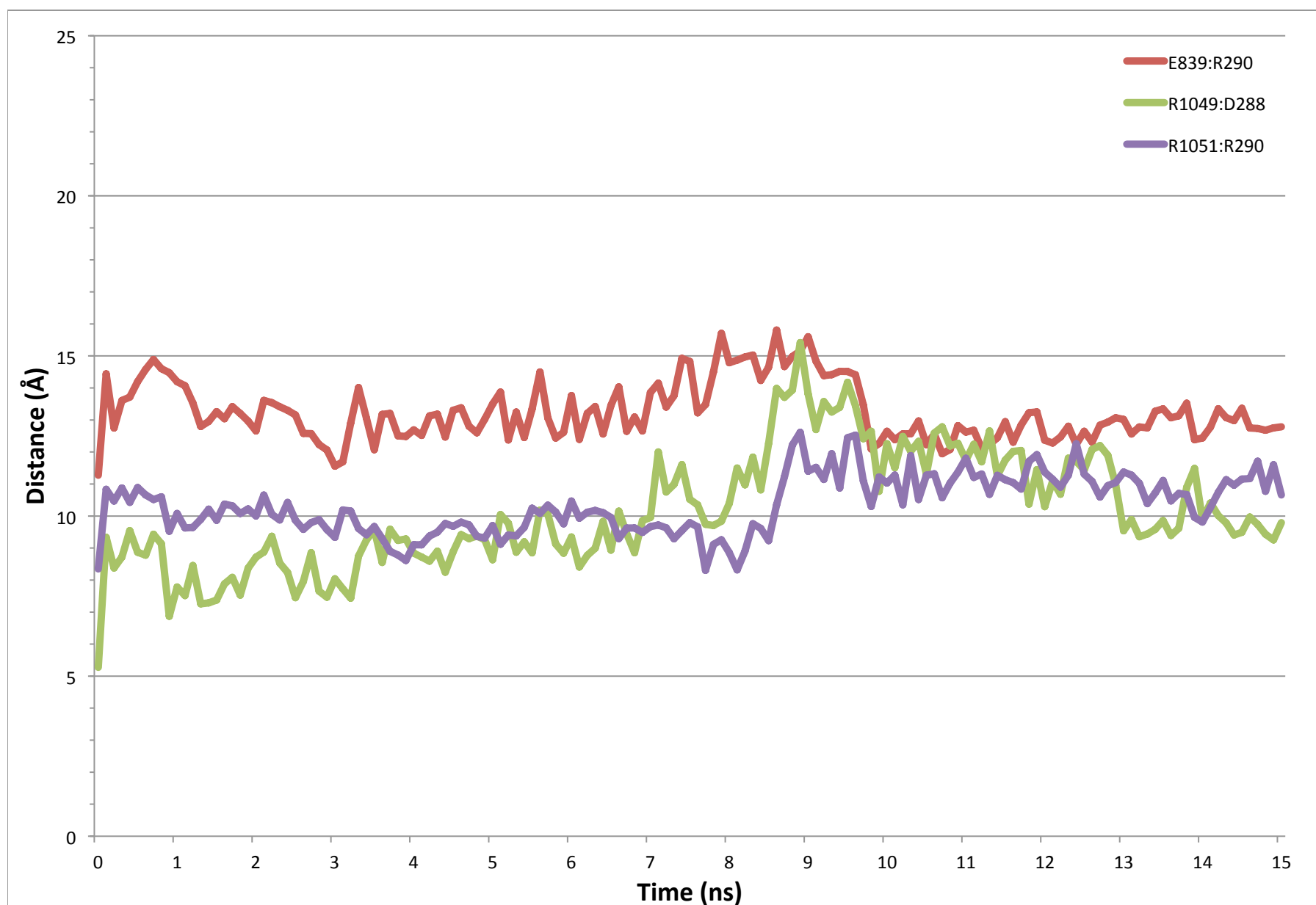

Supplement: Figure S10 — Distance between interacting residues on Vt (exposed) and S3 of the actin barbed-end. Simulation of Vt with the barbed-end of F-actin while approaching S3 with its exposed surface showed little linkage between Vt and F-actin. The distance between 3 Vt residues and their respective interacting residues in S3 are tracked and plotted. The Cα of all 3 residues are within 12 Å after 9 ns of simulation and are close enough to be associating. The linkages are not as strong as between Vt and S1. (PDF) [file pcbi.1002995.s010.pdf]

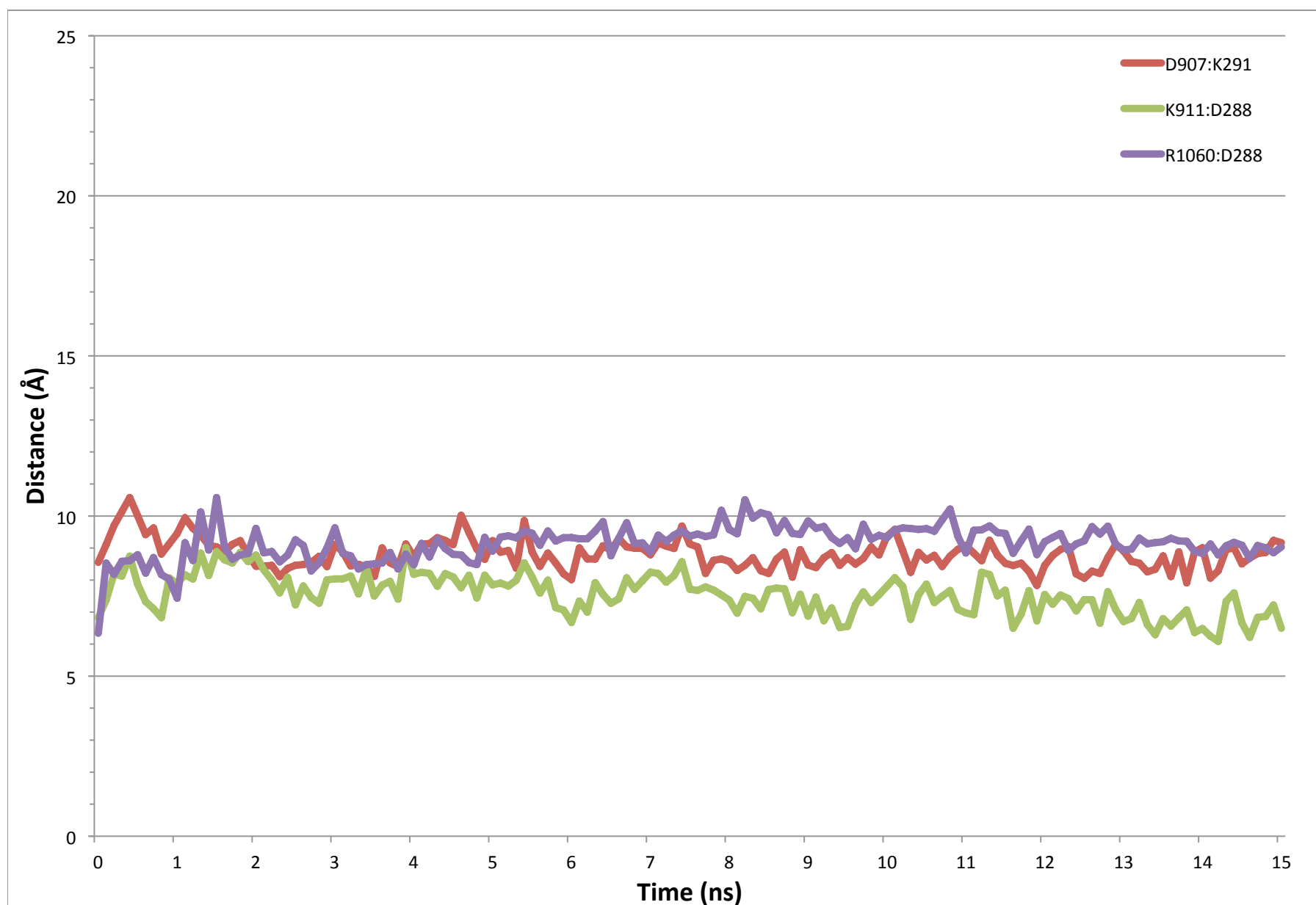

Supplement: Figure S11 — Distance between interacting residues on Vt (occluded) and S3 of the actin barbed-end. Simulation of Vt with the barbed-end of F-actin while approaching S3 with its occluded surface showed little linkage between Vt and F-actin. The distance between 3 Vt residues and their respective interacting residues in S3 are tracked and plotted. The three residues remain within 10 Å of each but do not associate any closer. (PDF) [file pcbi.1002995.s011.pdf]

Energy (kcal/mol)

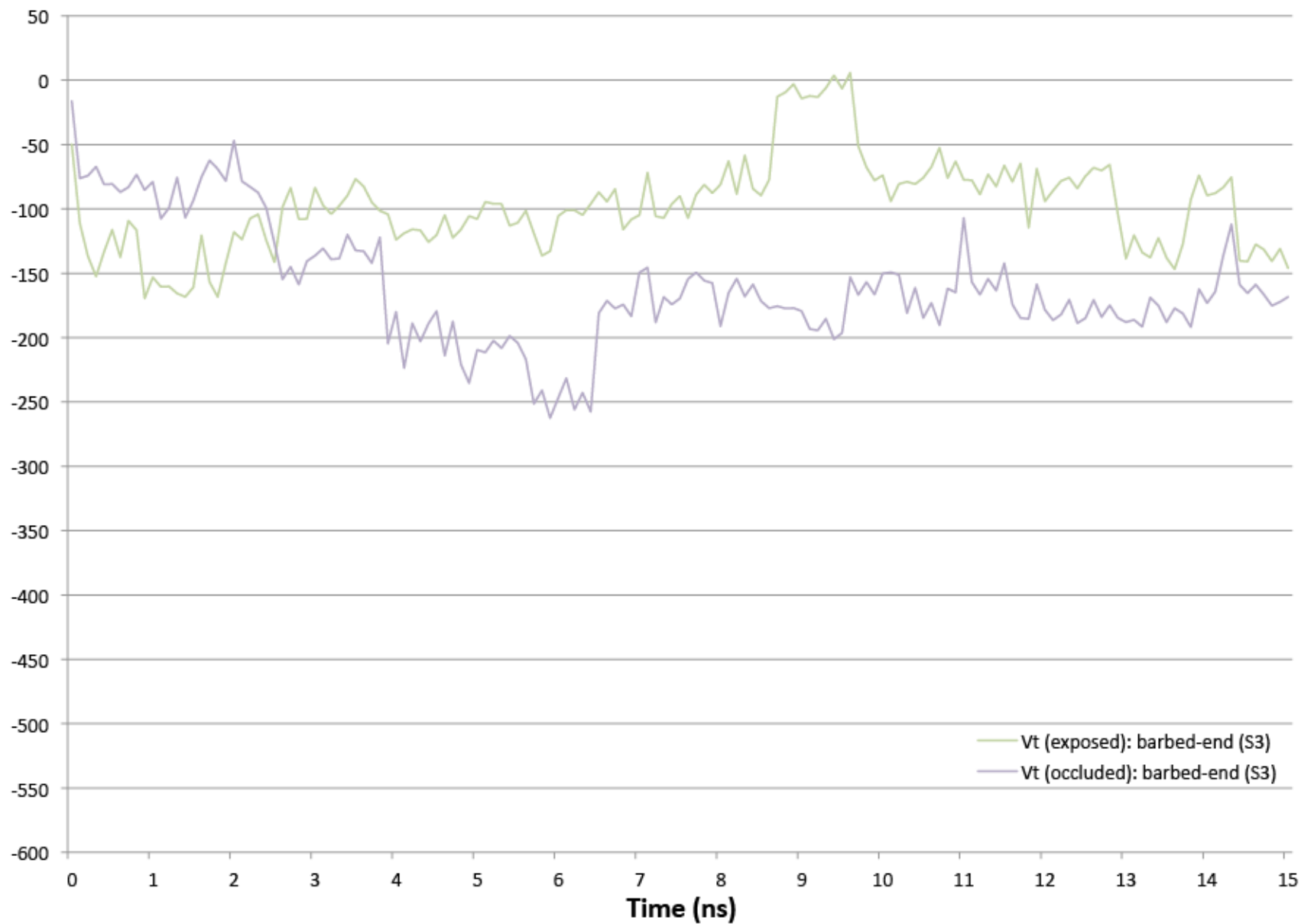

Supplement: Figure S12 — Comparison of potential energy changes from S1 capping by exposed and occluded Vt. The potential energy between binding residues in Vt and S1 of the capping-end of F-actin is calculated throughout the 15 ns simulations of both interaction with the exposed surface of Vt and interaction with the occluded surface of Vt. The potential energy of the exposed surface's interaction is plotted in green and the potential energy of the occluded surface's interaction is plotted in blue. Interaction with the exposed surface never reduces the potential energy between the binding residues more than 150 Kcal/mol whereas interaction with the occluded surface of Vt reduced the potential energy between the binding residues by as much as 250 Kcal/mol. Both interactions are favorable, but the interaction with the occluded surface is more stabilizing as it reduces the potential energy further. (PDF) [file pcbi.1002995.s012.pdf]

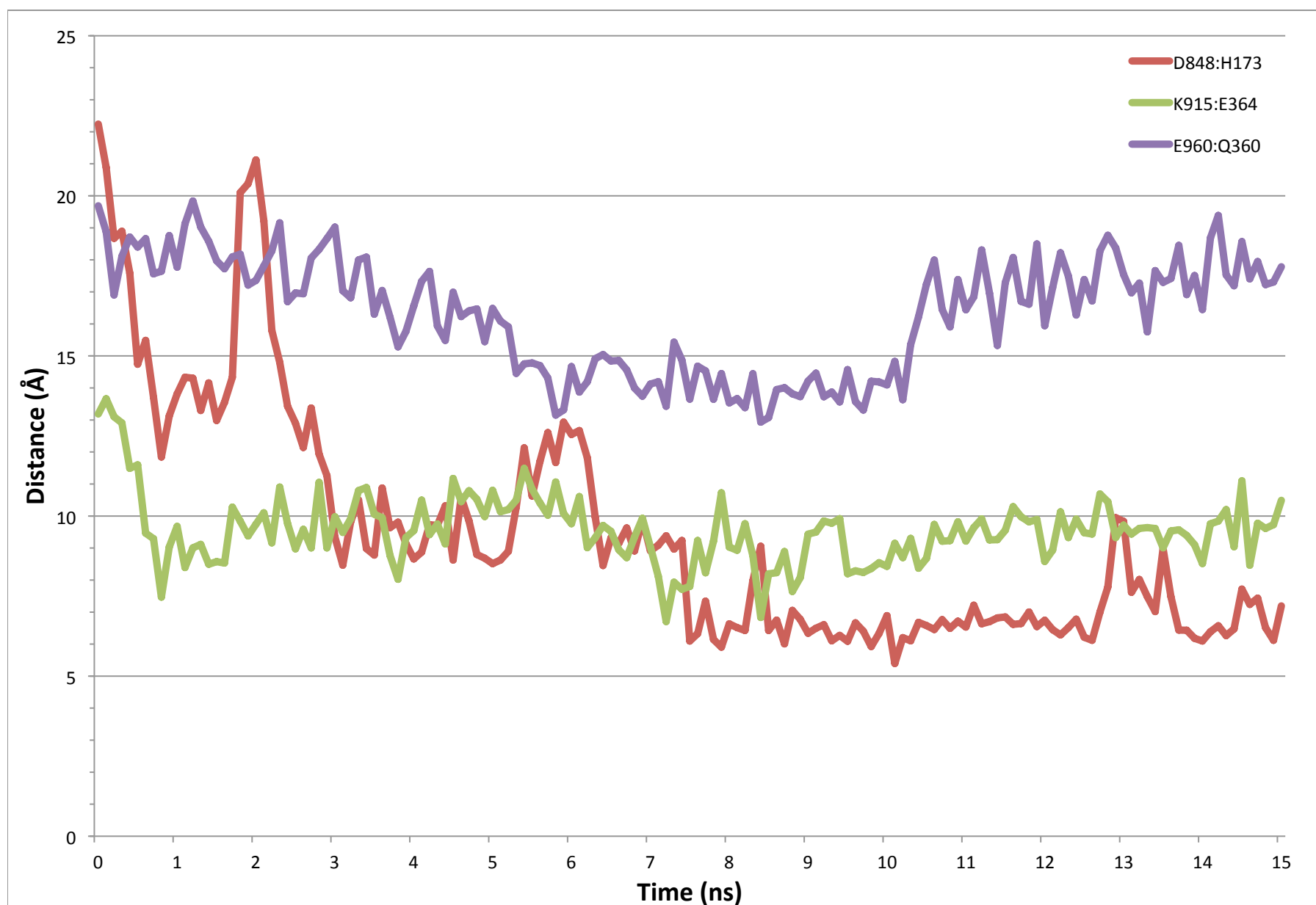

Supplement: Figure S13 — Distance between interacting residues on Vt (exposed) and both S1 and S3 of the actin barbed-end. Simulation of Vt with the barbed-end of F-actin while approaching both S1 and S3 with its exposed surface showed some linkage. The distance between 3 Vt residues and their respective interacting residues on S1 and S3 are tracked and plotted. Two interaction are stable and link Vt to both S1 and S3, one interaction, E960 and Q360, was intermittent and dissociated after 10 ns of simulation. (PDF) [file pcbi.1002995.s013.pdf]

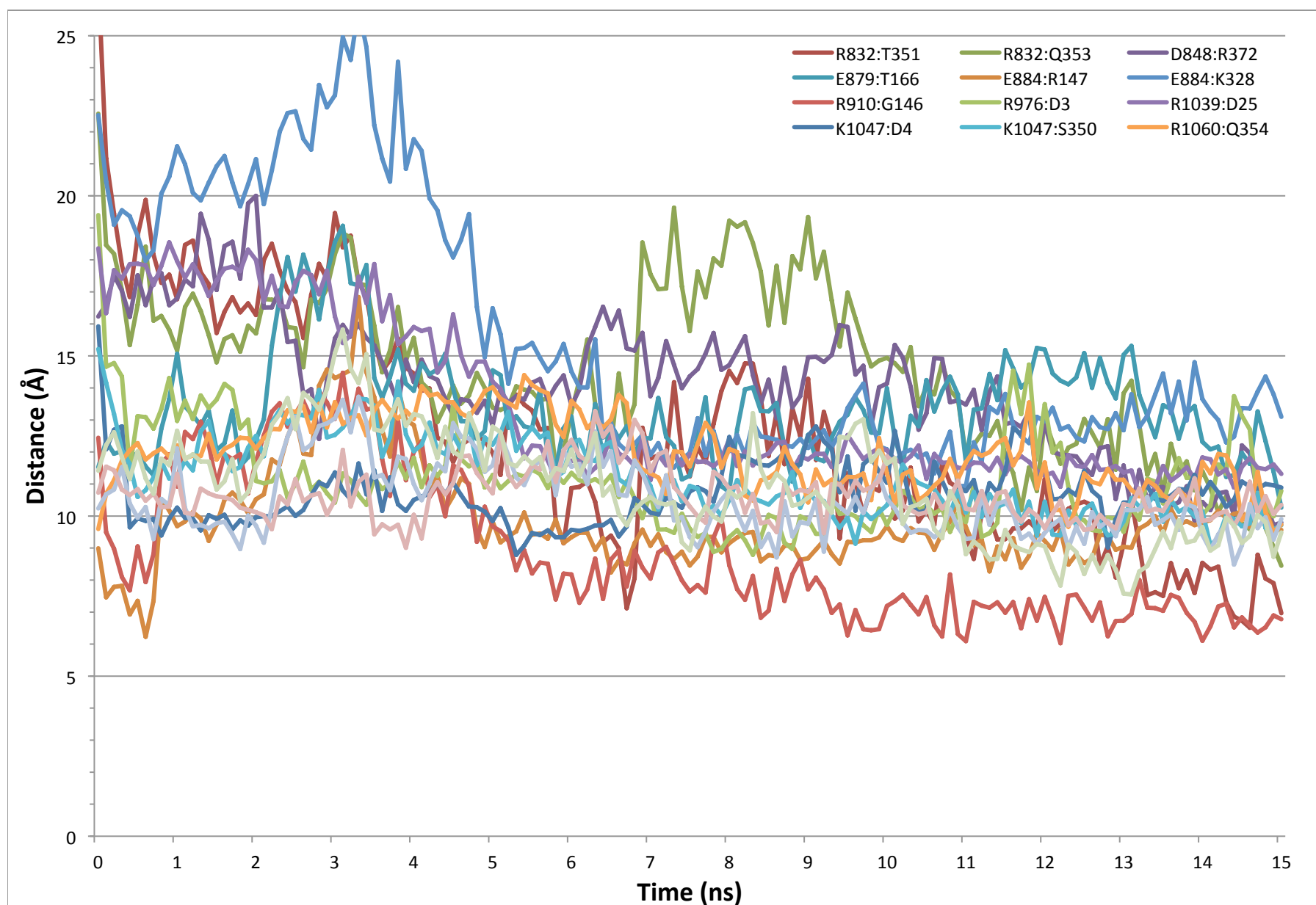

Supplement: Figure S14 — Distance between interacting residues on Vt (occluded) and both S1 and S3 of the actin barbed-end. Simulation of Vt with the barbed-end of F-actin while approaching both S1 and S3 with its exposed surface showed strong linkage. The distance between 12 Vt residues and their respective interacting residues on S1 and S3 are tracked and plotted. All 12 interactions showed stable linkage within 10 ns of simulation and remained linked for the remaining 5 ns of the simulation. This interaction was the strongest of the simulations of Vt interacting with the barbed-end of F-actin. (PDF) [file pcbi.1002995.s014.pdf]

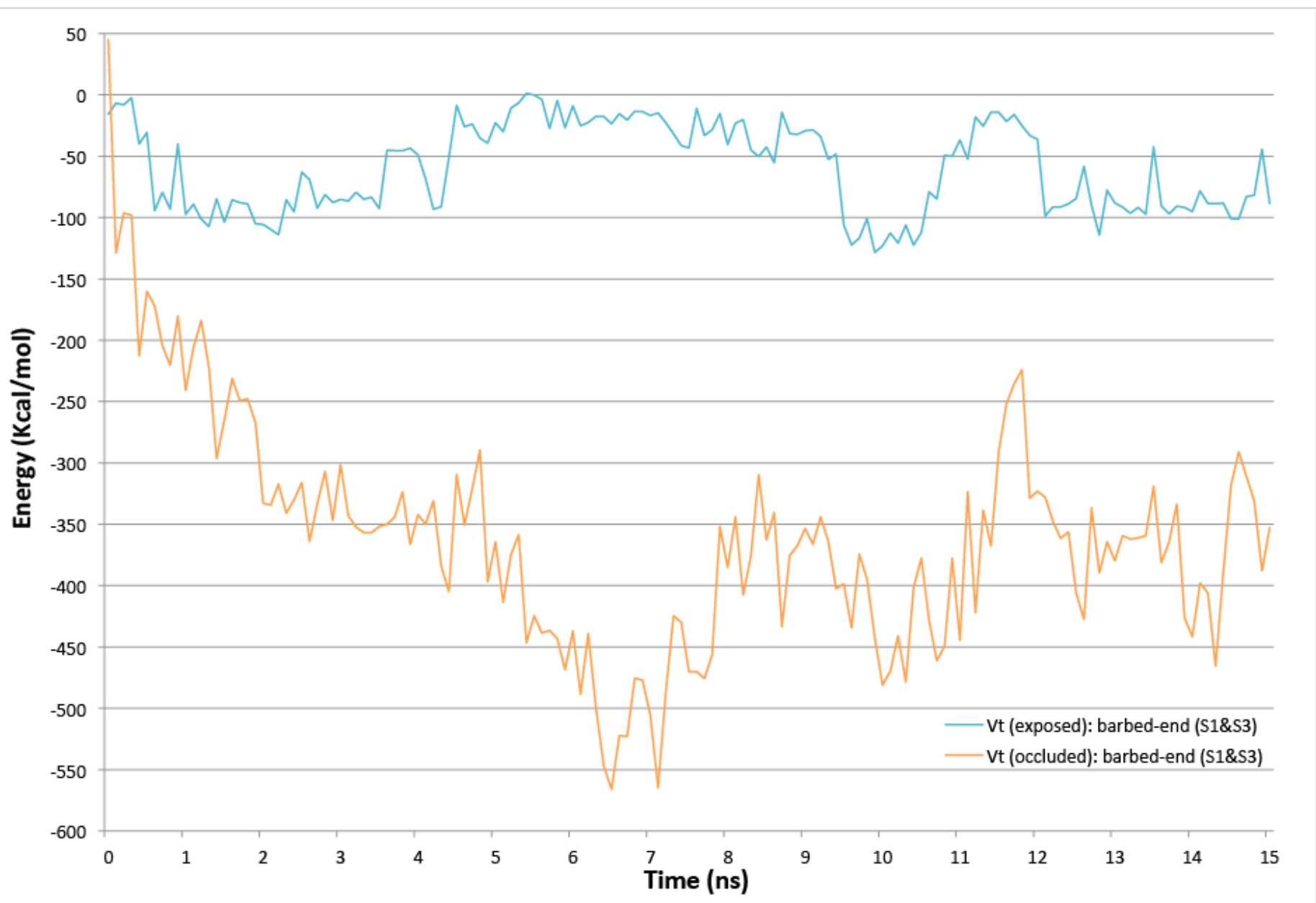

Supplement: Figure S15 — Comparison of potential energy changes from capping S1 and S3 by exposed and occluded Vt. The potential energy between binding residues from Vt and S1 and S3 of the barbed-end of F-actin is calculated throughout the 15 ns simulation for both the interaction of the exposed surface of Vt with S1 and S3 and the interaction of the occluded surface of Vt with S1 and S3. The interaction of Vt using its exposed surface with F-actin reduces the potential energy of the complex by 100 Kcal/mol. The interaction of Vt using it occluded surface with F-actin reduces the potential energy of the complex by over 550 Kcal/mol. The interaction between the occluded surface of Vt and the S1 and S3 subdomains is highly energetically favorable and likely represents the true binding interface between Vt and the barbed-end of F-actin. (PDF) [file pcbi.1002995.s015.pdf]

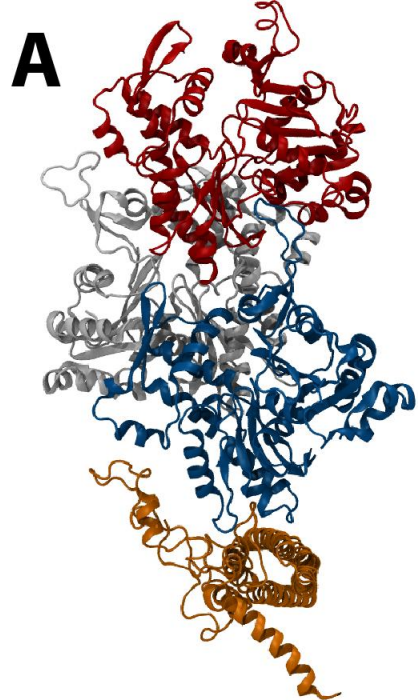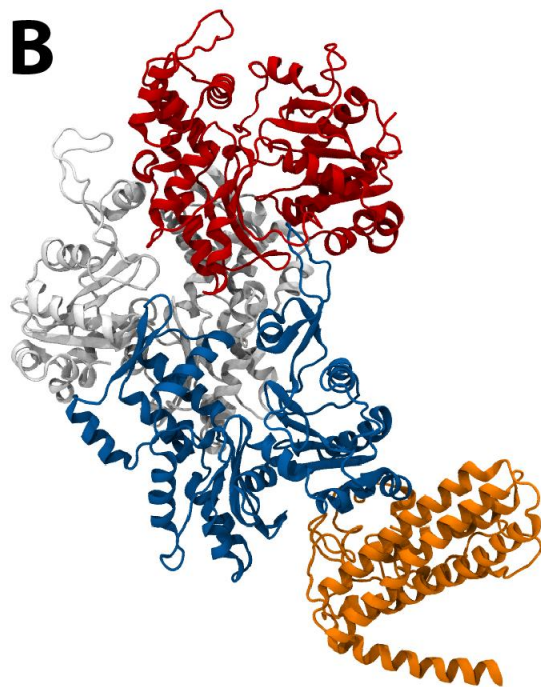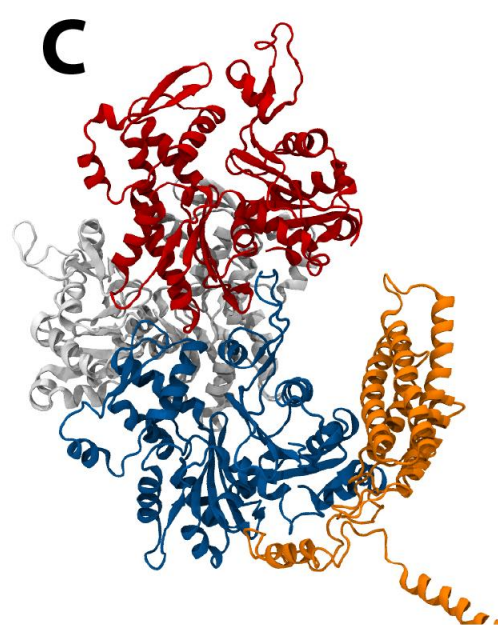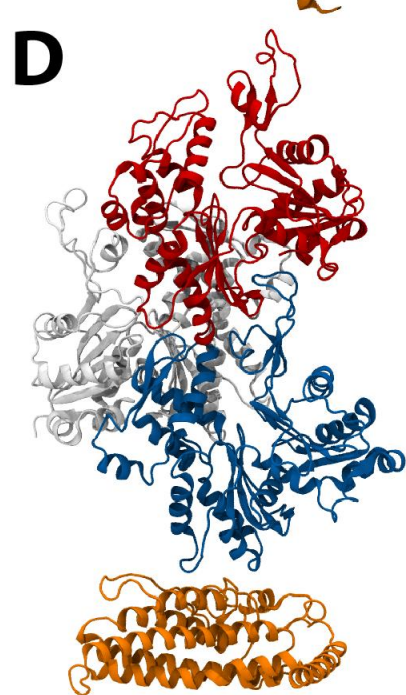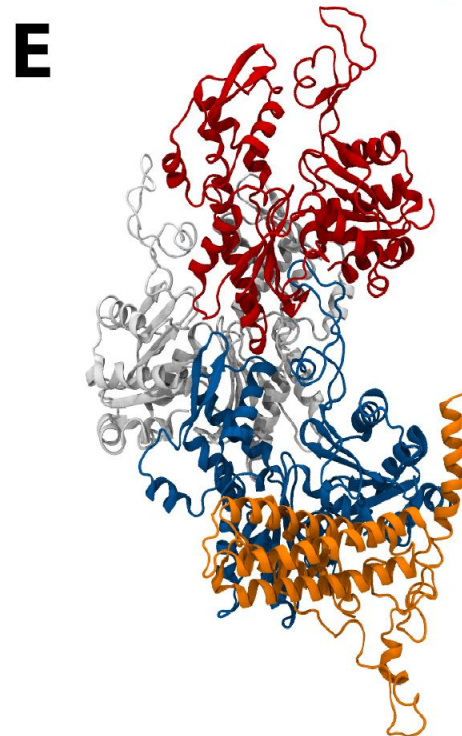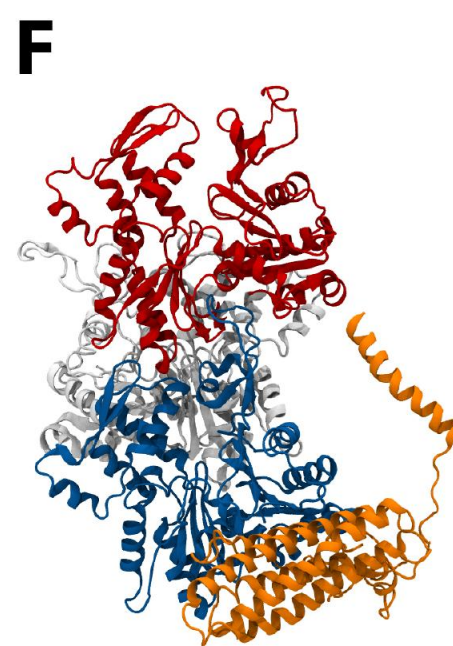

Supplement: Figure S16 — F-actin capping by Vt was investigated with six possible arrangements. A total of six possible arrangements of Vt and F-actin were simulated to investigate the interaction between vinculin and F-actin. Two surfaces of Vt were used, the surface exposed to solvent and F-actin (A), (B), and (C), and the surface normally occluded from F-actin by the vinculin head (D), (E), and (F). Both surfaces were initially arranged such that they were oriented towards S1 (C) and (F), towards S3 (A) and (D), or towards both S1 and S3 (B) and (E); exposed and occluded surfaces respectively. Interaction of Vt with either S1 only or with S1 and S3 was found to be more stable. Interaction with the occluded surface of Vt was found to be more likely than interaction with the exposed surface of Vt. Vt is shown in orange. Subunit n is shown in blue, and subunit n-2 is shown in red. (PDF) [file pcbi.1002995.s016.pdf]

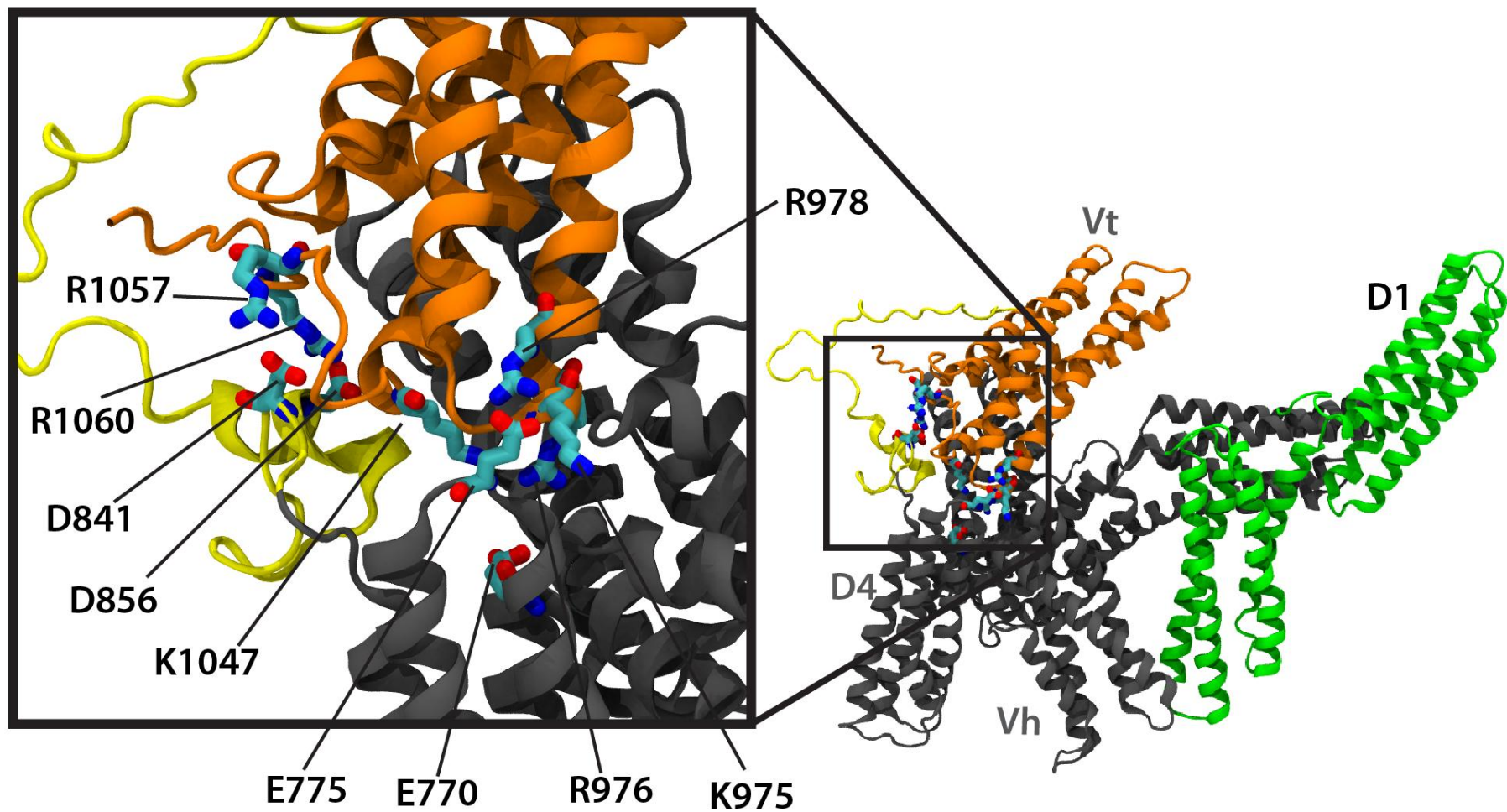

Supplement: Figure S17 — The interactions between D4 and Vt break to allow for the open II conformation. To produce the open II conformation, Vt is pulled away from D4. There are 5 salt-bridges between D4 and Vt: R1057 with D847, R1060 with D856, R978 with K1047, K975 with E775, and R976 with E770. The presence of these salt-bridges stabilized Vt near to the vinculin head and likely prevents interaction of Vt with the barbed-end of F-actin. After these interactions are broken and Vt separated from D4 and the rest of the vinculin head domain, then Vt is likely to be able to bind the barbed-end of the actin filaments. (PDF) [file pcbi.1002995.s017.pdf]

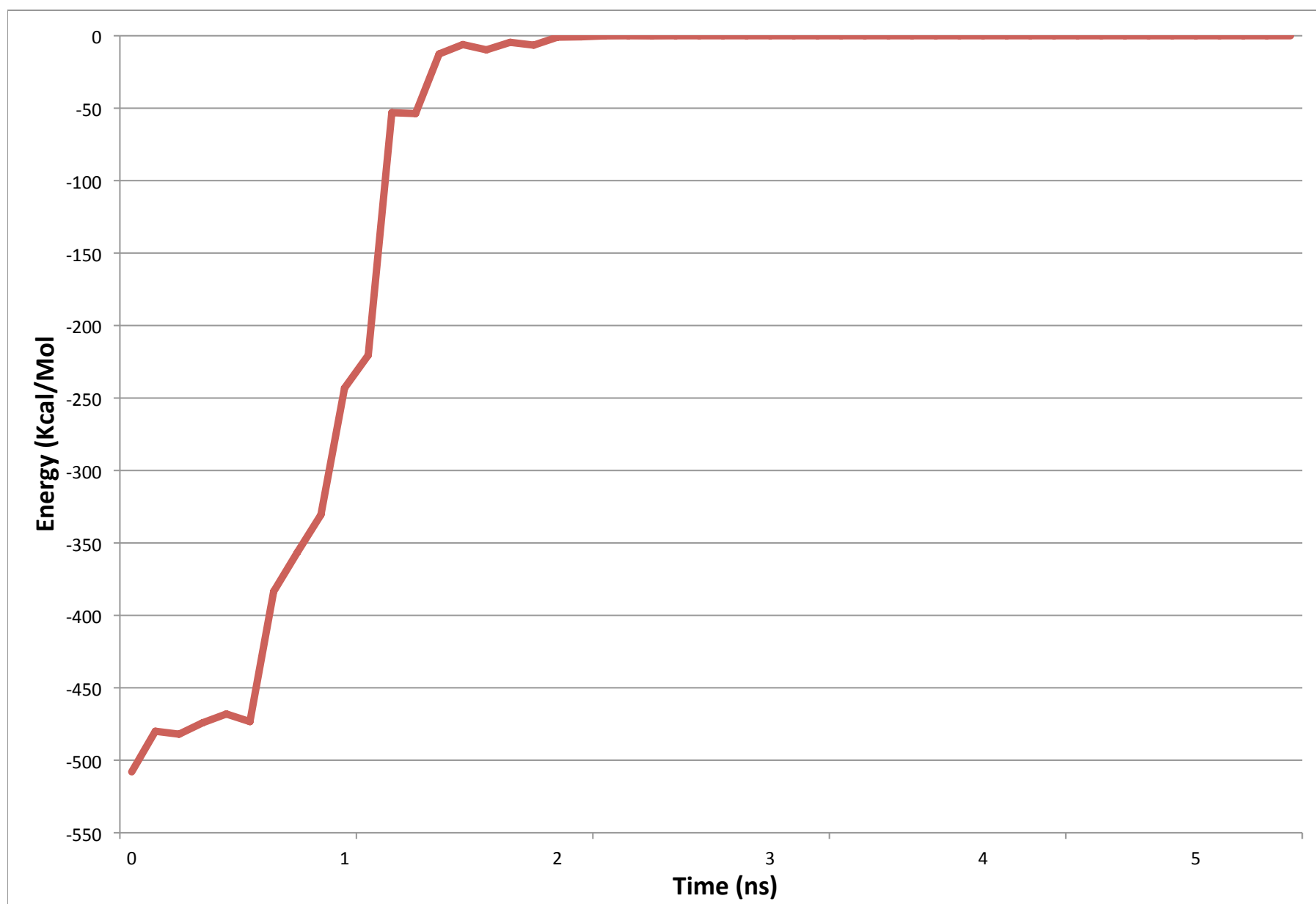

Supplement: Figure S18 — Potential energy between interacting residues in Vt and D4 during open II formation. The potential energy between residues interacting on Vt and D4 is calculated throughout the simulation of open II formation. The first 5 ns of the simulation are plotted here. The interactions included in the calculation are: R1057 with D841, R1060 with D856, K1047 and R976 with E770, R978 with E775, and K975 with E770. Initially there is over 500 Kcal/mol of potential energy stored in the interaction between Vt and D4. As Vt separates during formation of the open II conformation the stored energy is relieved. Within 2 ns of simulation the interactions are broken. (PDF) [file pcbi.1002995.s018.pdf]

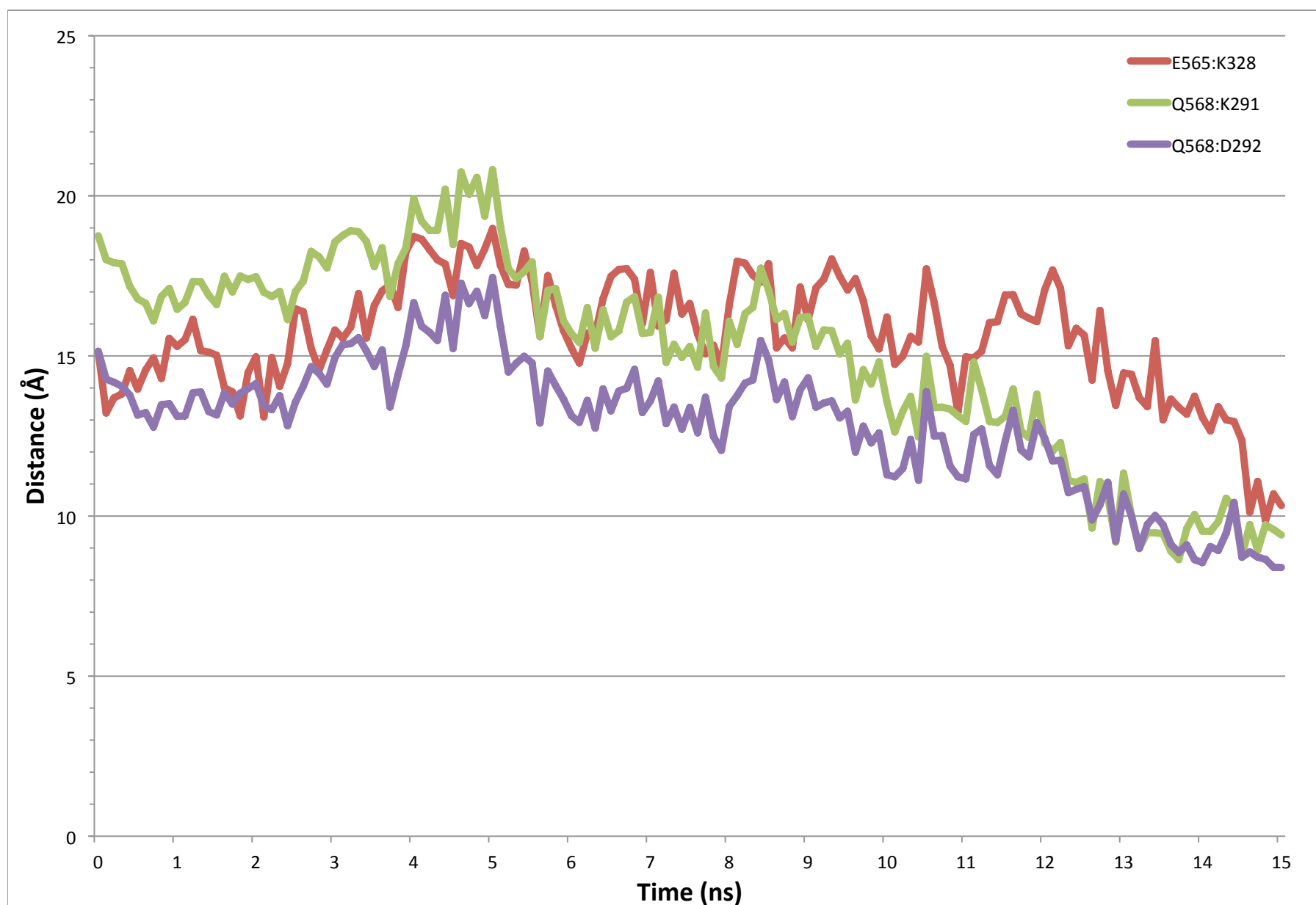

Supplement: Figure S19 — Distance between interacting residues on closed vinculin and F-actin. Simulation of full-length vinculin in its closed conformation with the barbed-end of F-actin while approaching both S1 and S3 showed little linkage. The distance between 3 residues on vinculin and their respective interacting residues on S1 and S3 are tracked and plotted. All 3 interactions did interact favorable. By the end of the 15 ns of simulation they are closer than 10 Å from each other. (PDF) [file pcbi.1002995.s019.pdf]

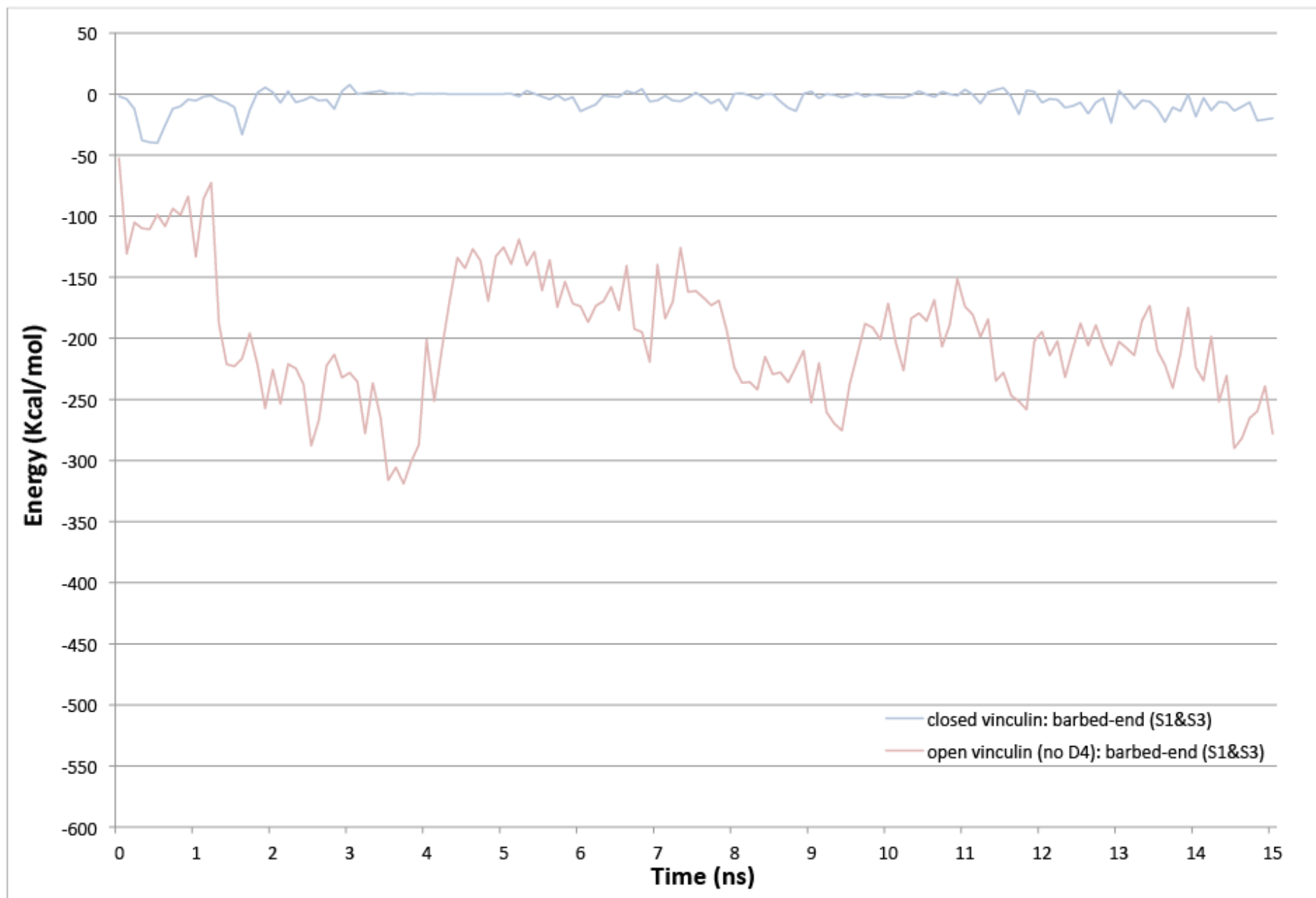

Supplement: Figure S20 — Comparison of the potential energy change after capping by two vinculin conformations. The potential energy between interacting residues on vinculin and the barbed-end of F-actin are calculated throughout 15 ns of simulation for both simulation with the closed conformation and simulation with a conformational change releasing Vt from the vinculin head. The potential energy between interacting residues is never reduced more than 50 Kcal/mol in interaction with the closed conformation of vinculin (blue plot). In contrast, interaction of vinculin after a second conformational change with S1 and S3 reduces the potential energy of the interacting residues by as much as 300 Kcal/mol. This interaction is highly energetically favorable and likely represents the true interface formed by capping of F-actin by full-length vinculin. (PDF) [file pcbi.1002995.s020.pdf]

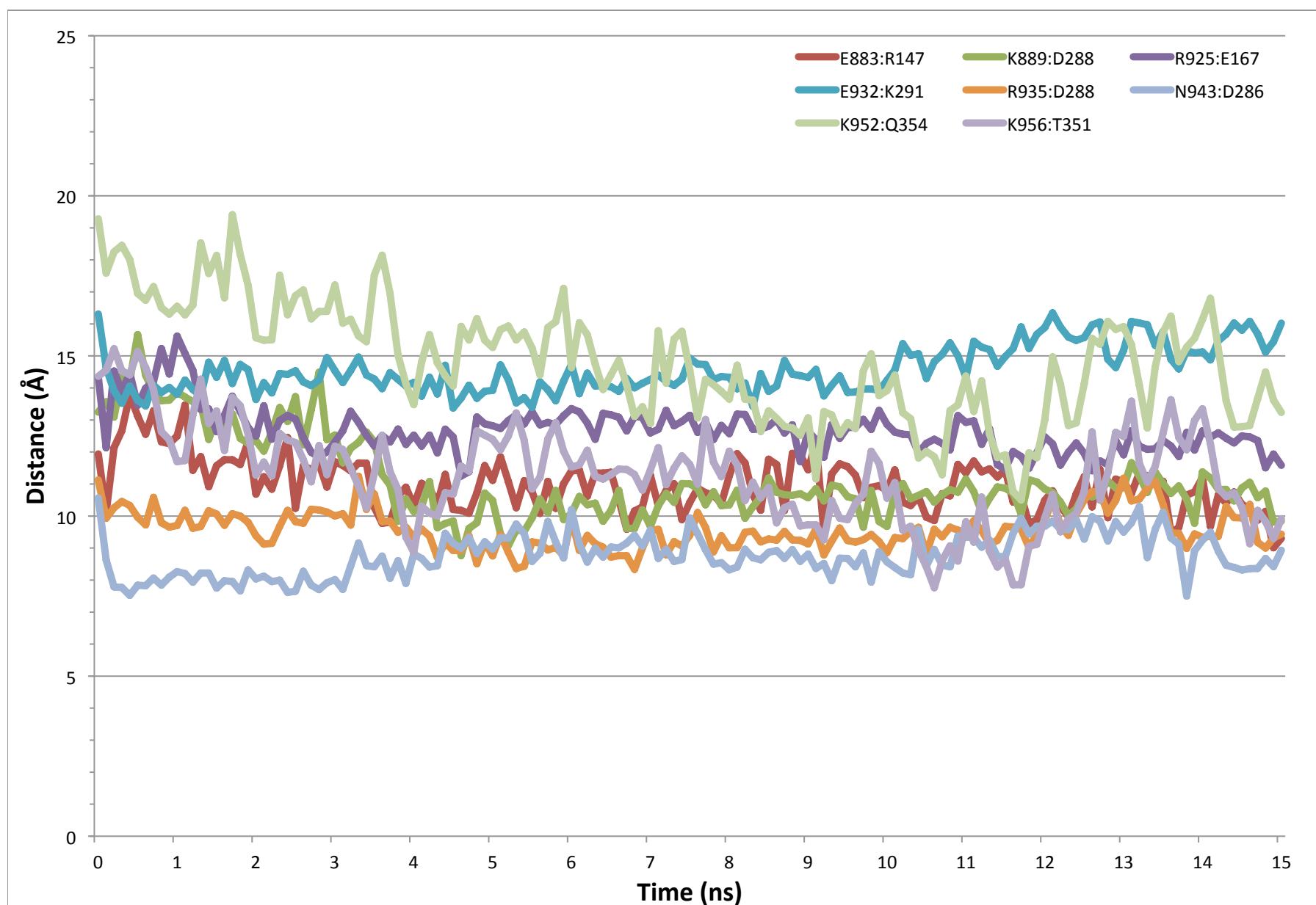

Supplement: Figure S21 — Distance between interacting residues on open II vinculin and F-actin. Simulation of full-length vinculin in its open II conformation with the barbed-end of F-actin while approaching both S1 and S3 showed strong linkage. The distance between 8 residues on Vt and their respective interacting residues on S1 and S3 are tracked and plotted. All 8 interactions show a linkage and a decrease in distances. The decrease in distances are not as large as with simulation of Vt alone. (PDF) [file pcbi.1002995.s021.pdf]
